# Diversification and post-glacial range expansion of giant North American camel spiders in genus *Eremocosta* (Solifugae: Eremobatidae)

**DOI:** 10.1038/s41598-021-01555-1

**Published:** 2021-11-11

**Authors:** Carlos E. Santibáñez-López, Paula E. Cushing, Alexsis M. Powell, Matthew R. Graham

**Affiliations:** 1 Department of Biology, Eastern Connecticut State University, 83 Windham Street, Willimantic, CT 06226 USA; 2 Department of Biological and Environmental Sciences, Western Connecticut State University, 181 White Street, Danbury, CT 06810 USA; 3 Department of Zoology, Denver Museum of Nature and Science, 2001 Colorado Boulevard, Denver, CO 80205 USA

**Keywords:** Evolutionary biology, Population genetics

## Abstract

Species of camel spiders in the family Eremobatidae are an important component of arthropod communities in arid ecosystems throughout North America. Recently, research demonstrated that the evolutionary history and biogeography of the family are poorly understood. Herein we explore the biogeographic history of this group of arachnids using genome-wide single nucleotide polymorphism (SNP) data, morphology, and distribution modelling to study the eremobatid genus *Eremocosta*, which contains exceptionally large species distributed throughout North American deserts. Relationships among sampled species were resolved with strong support and they appear to have diversified within distinct desert regions along an east-to-west progression beginning in the Chihuahuan Desert. The unexpected phylogenetic position of some samples suggests that the genus may contain additional, morphologically cryptic species. Geometric morphometric analyses reveal a largely conserved cheliceral morphology among *Eremocosta* spp. Phylogeographic analyses indicate that the distribution of *E. titania* was substantially reduced during the last glacial maximum and the species only recently colonized much of the Mojave Desert. Results from this study underscore the power of genome-wide data for unlocking the genetic potential of museum specimens, which is especially promising for organisms like camel spiders that are notoriously difficult to collect.

## Introduction

The arachnid order Solifugae, also known camel spiders, is a poorly studied group of mostly nocturnal predatory arachnids with powerful chelicerae and voracious appetites^1^. They are distributed throughout a variety of habitats globally but are particularly diverse in arid ecosystems where they are important predators of arthropods^2^. Solifugae currently comprises about 1095 species found on all continents except Antarctica and Australia^3,4^. Despite their diversity, abundance, and widespread occurrence, little is known about how they diversified and radiated into different niches across the globe. Fortunately, methodologies are improving^5,6^ and progress is being made with one group, the North American family Eremobatidae Kraepelin, 1899.

Eremobatidae spp. occur throughout western North America where they are common elements of arid ecosystems. Multilocus data from 81 exemplar taxa, by far the most comprehensive phylogenetic analysis of camel spiders to date, revealed that much of our understanding about relationships among eremobatid species is flawed, necessitating an urgent need for taxonomic revision^7^. Molecular clock estimates from the same study indicate that Eremobatidae is nearly as old as the North American deserts it inhabits, and that geologic activity and climate fluctuations associated with desert formation probably facilitated diversification.

**Figure 1:**
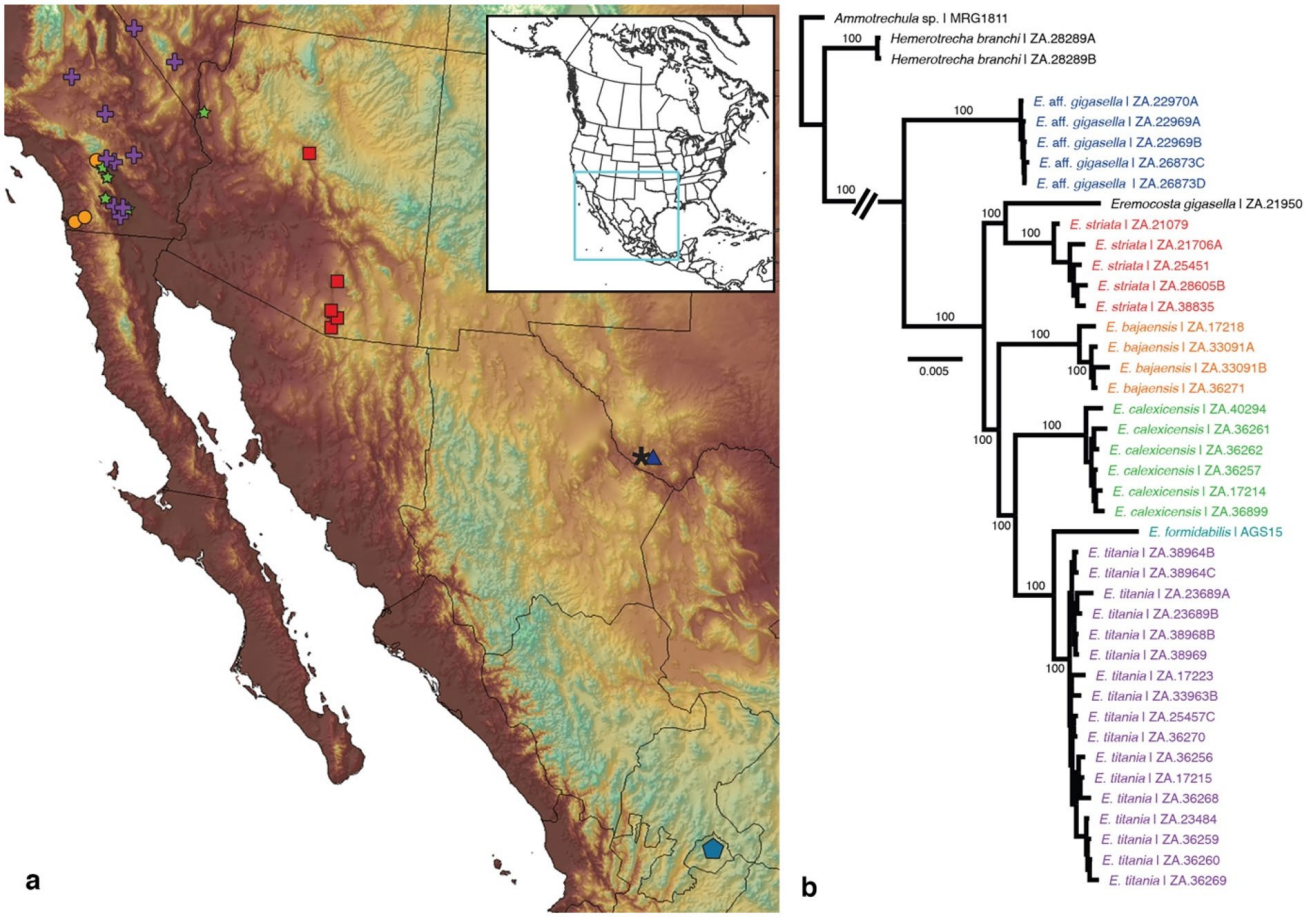
(**a**) Map showing locations of *Eremocosta* samples used in this study. Cyan pentagon: *E. formidabilis*. Blue triangle: *E.* aff. *gigasella*. Black asterisk: *E. gigasella*. Red square: *E. striata*. Orange circle: *E. bajaensis*. Green star: *E. calexicensis*. Purple cross: *E. titania*. (**b**) Maximum likelihood tree topology recovered from the analysis of 42 solifugid samples and 521,343 sites using the GTR + F + R2 model as selected by ModelFinder (m21; lnL = − 908,151.1928). Numbers on nodes indicate ultrabootstrap support. Nodes without numbers were supported by less than 100%. Distribution map was generated in ArcGIS v. 10.1 (ESRI, Redlands, CA, USA) using the locality coordinates, a base map from ArcGIS and a digital elevation model from DIVA-GIS, available at http://www.diva-gis.org/Data.

We used genome-wide single nucleotide polymorphism (SNP) data to study the giant camel spiders of *Eremocosta* Roewer 1934. Members of the genus grow to over 50 mm, exceptionally large for eremobatids. *Eremocosta* consists of seven species that occupy some of the most extreme of North America’s desert environments and recently underwent a taxonomic revision^8^. These species are broadly distributed, ranging from southern and central Mexico throughout the western United States (Fig. 1A). This distribution spans several biogeographic barriers that have had different levels of influence on desert taxa, such as the Salton Trough, Colorado River, and Cochise Filter Barrier^9–11^.

Our main objectives in this study were to (1) resolve relationships among *Eremocosta* spp., (2) reconstruct the order, timing, and potential drivers of diversification, and (3) assess the impact of Pleistocene climate fluctuations on camel spiders by conducting a phylogeographical analysis of an individual *Eremocosta* species. Given the power of genome-wide SNP data, we also assessed the direction and magnitude of gene flow among populations of the species *Eremocosta titania*. Additionally, we used a geometric morphometric analysis of *Eremocosta* chelicerae to determine if colonization of different areas was coupled with morphological diversification. Results from this study add another layer to our understanding of accumulation and maintenance of arid-adapted biodiversity in the deserts and semi-deserts of North America.

## Results

### Matrices and phylogenies

High-throughput sequencing of ddRAD libraries generated a total of 353,916,765 reads obtained from 68 solifugids individuals (mean = 5,204,658, SD ± = 8,260,842). Two samples did not pass the assembly filters and were omitted. Our first assembly comprised of loci shared by at least 17 samples consisted of 25,092 loci, with 64% of missing data (Table S1). Preliminary ML phylogenies using this matrix (not shown) recovered some inconsistency in the monophyly of some species. Therefore, new assemblies were conducted that excluded 24 samples with high amounts (> 95%) of missing data. Details about these matrices are summarized in Table S1.

ML analyses using the SNPs, the unlinked SNPs (uSNPs) and the full matrices of loci shared by at least 21 samples, rendered *Eremocosta* as monophyletic with strong support (Fig. 1B, Fig. S1). Similarly, each *Eremocosta* species except *E. gigasella* was monophyletic with 100% support. All major nodes within the genus received 100% support.

Five of the *Eremocosta gigasella* were consistently recovered as a clade sister to all other remaining species. The remaining sample (DMNS ZA.21950) grouped with *E. striata* with strong support but produced a long branch*.* All of the *E. gigasella* samples were collected near the Dalquest Desert Research Station in the northern Chihuahuan Desert, but in different habitats. The sample sister to *E. striata* was collected from up on a plateau, whereas the five divergent samples were found in adjacent canyonlands. Interestingly, a new species of solifuge genus *Chambria* was discovered in these canyons (PEC, unpublished), as well as a myrmecophilic spider that represents a new family^12^. Given these patterns, we suspect the sample that was sister to *E. striata* is true *E. gigasellae*, and that the others likely represent a new species*.* Additionally, our single sample of *E. formidabilis* was recovered as sister to *E. titania* with strong support.

### Divergence dating and ancestral area reconstructions

Our re-analysis of the four-gene data from Cushing et al.^7^, but using a more typical rate calibration for arthropods, yielded a topology that was largely congruent. The mean time to the most recent common ancestor (TMRCA) of extant Eremobatidae was estimated to be in the Miocene (18 Ma). *Eremocosta* was estimated to have diversified beginning in the late Miocene and early Pleistocene (Fig. S2). Our analysis of RADseq data calibrated with older dates from Cushing et al.^7^ estimated the divergence of crown *Eremocosta* to have occurred during the mid to late Miocene with a mean of 11 Ma (95% HPD = 5–18 mya, Fig. S3). When calibrated with younger dates, the TRMCA for *Eremocosta* estimated to be in the late Miocene to early Pleistocene, with a mean of 6 Ma (95% HPD = 5–8 Ma; Fig. S4).

**Figure 2:**
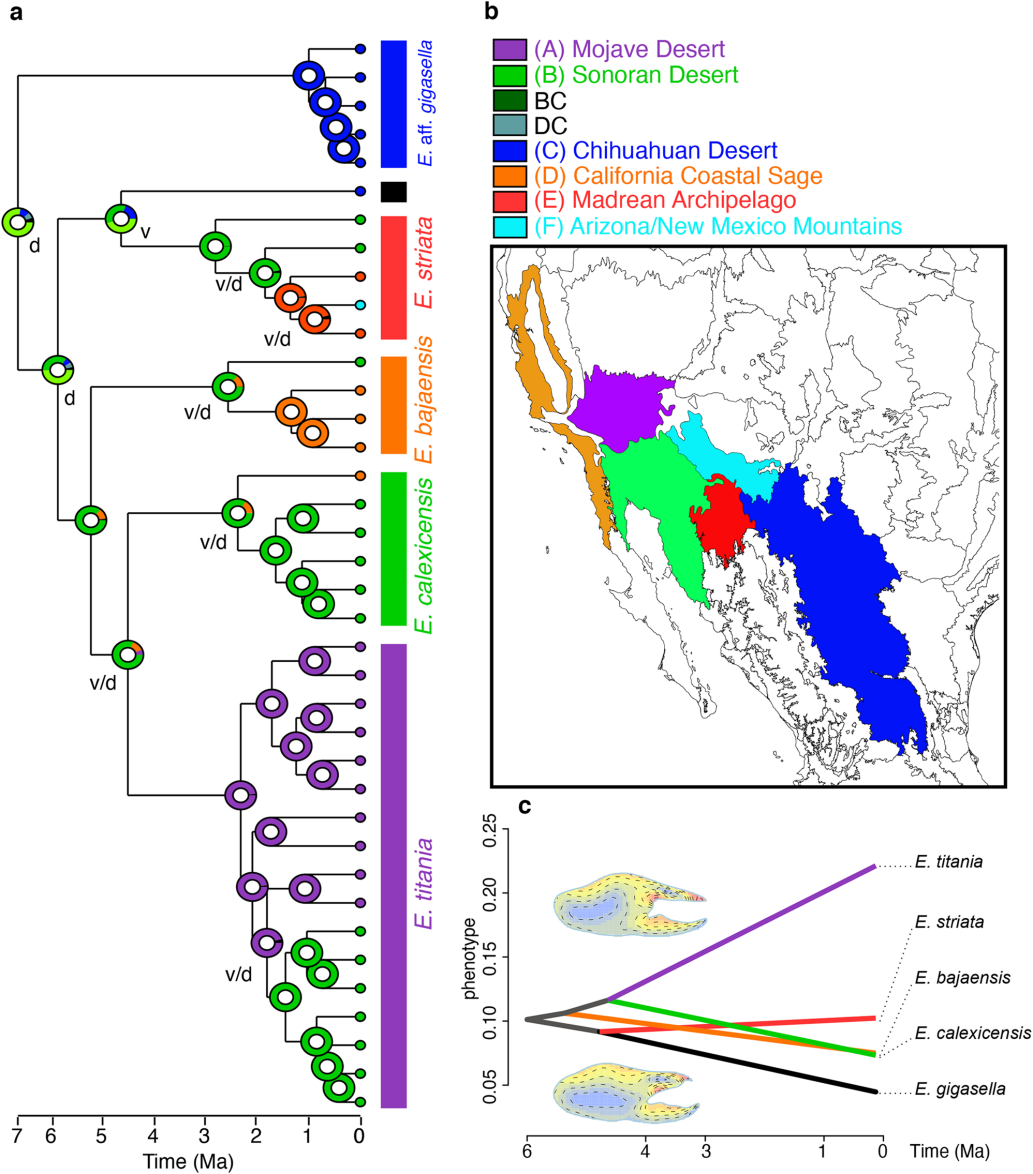
Historical biogeography of the camel spider genus *Eremocosta*. Ancestral areas estimated by RASP using *DEC* + *j* were mapped on MRCA nodes for each species in our favored time-calibrated topology (**a**) and onto the ecoregions map of North America (**b**). The colors of each circle indicate the proportion of support for each biogeographical area received for that node. Dispersal = d, vicariance = v and both = v/d events were indicated on each node. (**c**) Visualization of the Euclidean distances as a measure of the variation of morphology in the sexual dimorphism of male and female chelicerae (dextral surface) onto our dated molecular tree; x-axis indicates the time of divergence; y-axis indicates the Euclidean distance. Superimposed chelicerae (as drawn by *momocs*) showing the heat map on most important changes between both sexes: delimited chelicerae represent the one from male, whereas the colored represents the female (top); or delimited chelicerae represents the one from female and the colored represents the male (bottom). * = *E. gigasella*. Ecoregion map was generated in ArcGIS using a base map (Ecoregion level III) from the United States Environmental Protection Agency (EPA) (available at: http://epa.gov/eco-research/ecoregions-north-america).

Ancestral area reconstructions using the arthropod rate-calibrated chronogram and optimal model (DEC + j) suggested two areas (the Chihuahuan and Sonoran deserts) as the ancestral range for the genus (Fig. 2). Similarly, the second-best model (DIVALIKE + j) recovered the same combination of the Chihuahuan and Sonoran deserts as the ancestral range for the genus (Fig. S5). Both analyses suggested that common ancestor of *E. striata* colonized the Madrean Archipelago around 2 Ma, with divergence of *E. bajaensis* in Californian coastal sage habitats at about the same time. Both models suggest that *E. titania* colonized the Mojave Desert about 2 Ma prior to inhabiting the Sonoran Desert (Fig. 2).

**Figure 3:**
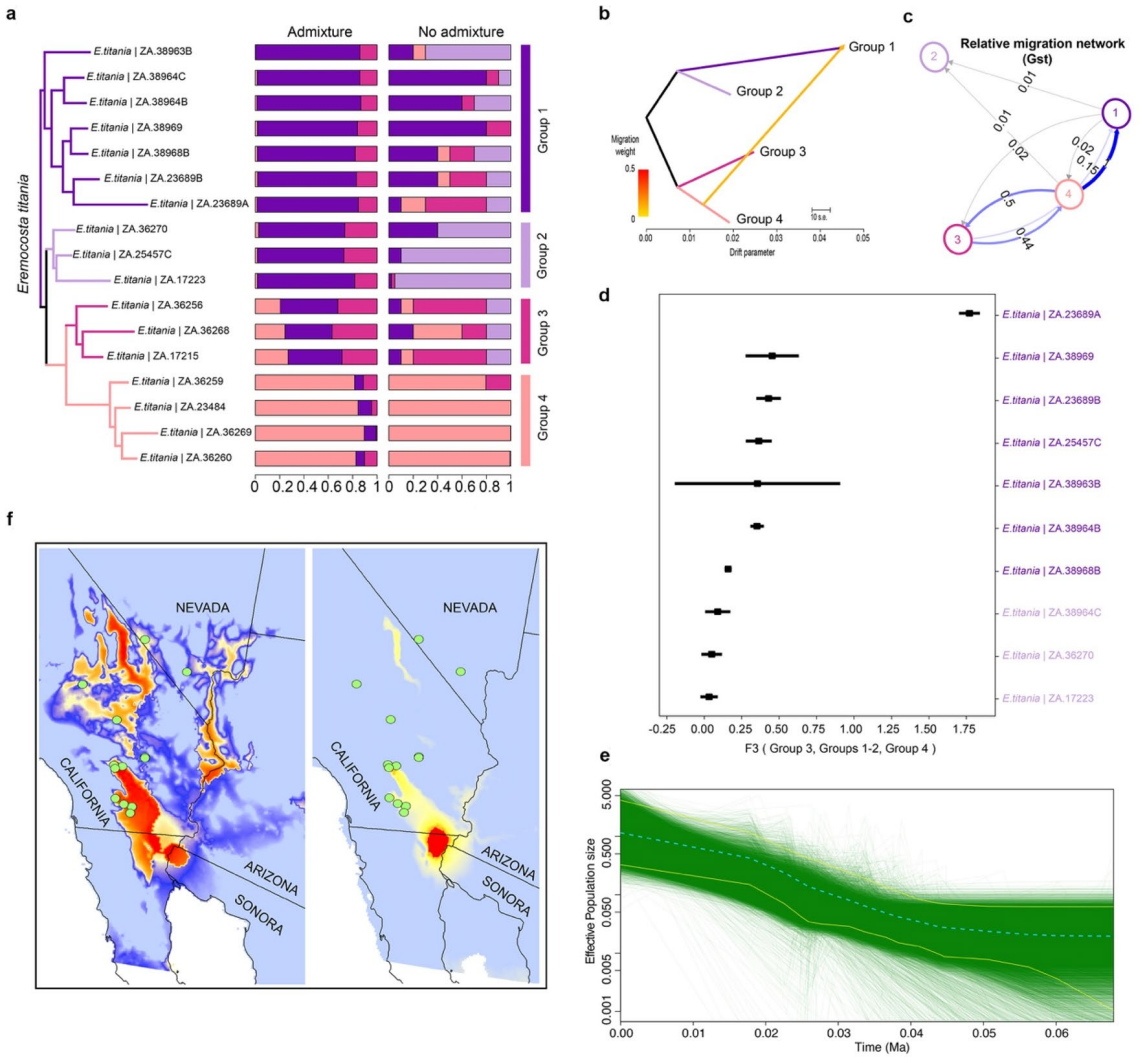
Population genomics and migration events within *E. titania*. (**a**) ML RADseq phylogeny with the 17 SNPs of *E. titania* and six of *E. calexicensis* (as outgroup, not shown), and 65,674 nucleotide sites using the GTR + F + R2 model as selected by ModelFinder (Ln = − 292,482.2174). Bar plots indicate result of genetic assignments based on the Bayesian method implemented in the software Structure using the 4207 uSNPs and the admixture or the no admixture model. (**b**) Visualisation of migration from population 4 towards population 1 as a measure of genetic drift, implemented in a treemix graph considering 10k and an unrooted topology. (**c**) Relative migration network calculated by the divMigrate function from the R package diveRsity selecting the *Gst* statistics. (**d**) Three-way admixture between Groups 1 or 2 and 3,4; negative values indicate that member (colored coded as its corresponding population) is admixed between populations 3 and 4. (**e**) Extended Bayesian Skyline Plot (EBSP) showing the dynamic population size for *E. titania* based on 4207 uSNPs. The y-axis indicates an effective population size scaled by mutation rate as a function of time. Light blue dotted line indicates median EBSP estimate, green lines show individual population trajectories, with the 95% highest posterior density limits shown in magenta. (**f**) Current (left) and late glacial (right) species distribution models showing the distribution of suitable (warm colors) and unsuitable (cool colors) climates for *E. titania* (map base from ArcGIS, and a political state division retrieved from DIVA-GIS, available at http://www.diva-gis.org/Data).

### Testing for the evolution of sexual dimorphism

In our morphometric analysis using the elliptic Fourier analysis (EFA) of the prolateral shapes in male chelicerae, PC1 explained 67% of the variation, with shapes on this component ranging from a slender (*E. striata*) to a rounder cheliceral manus, and the presence of more distinctive movable finger teeth (*E. bajaensis*, *E. gigasella*, and *E. calexicensis*; Fig. S6). PC2 explained less than 30% and separated chelicerae that exhibit a dorsal “hump” (a declivity between the dorsal surface of the manus down to the fixed finger; e.g. *E. calexicensis* and *E. gigasella*; Fig. S6). In the EFA of the prolateral shapes in female chelicerae, PC1 explained 86% of the variation and segregated the chelicerae of *E. titania* (Fig. S7). When PC1 was plotted onto the ML phylogeny (Fig. S7), female chelicerae did not change much until the morphology of common ancestor of *E. calexicensis* and *E. titania* diverged from the others. The morphology of the female chelicerae of *E. titania* then continued to diverge and is now significantly different (strong phenotype with a more globular cheliceral manus and wider/deeper cheliceral fingers as shown in Fig. S6) from the other four species studied. The morphology of male chelicerae exhibited a different pattern, with early morphological divergence with *E. striata* and later divergence of *E. titania*. Taken together, the chelicerae morphology is unique in both sexes for *E. titania* and in male *E. striata*.

The MANOVA comparing the sexual dimorphism recovered no significant difference between the outlines of both sexes (F_1,8_ = 8.53, *P* = 0.10). However, the Thin Iso Splines comparison of mean shape between the chelicerae of both sexes showed their strongest differences at the dorsal “hump” in male chelicerae, and the depth and margins of the movable and fixed fingers (Fig. 2c, Fig. S7). Euclidean distances plotted on the dated topology indicate *Eremocosta* spp. do not show strong dimorphism, with the exception of *E. titania* (with an EA > 0.25; Fig. 2c).

### Population structure—*E. titania*

Maximum likelihood analyses of matrices e18_SNPs and e13_SNPs recovered the presence of two clades within *E. titania* with strong support (Fig. 3A, Fig. S8). One clade contained all samples from the Mojave Desert, whereas a larger clade grouped samples from the Sonoran Desert. This Sonoran Desert clade was subdivided into three subclades (with 100% ultrabootstrap support values) in agreement with their distribution areas (Fig. 3A, Fig. S8). Similarly, the structure analysis using uSNPs (e13_uSNPs) with admixture found that a *K* = 3 was optimal, and divided *E. titania* into three distinct genetic clusters. In contrast, the structure analysis using no-admixture found that a *K* = 4 was optimal, subdividing *E. titania* into four clusters, which partially agrees with our ML topology (Group 1–4; Fig. 3A). In both analyses, only the genetic composition of Group 4 was inconsistent with the clades recovered as monophyletic in our ML topology. The genetic composition of Group 2, on the other hand, agreed with one monophyletic clade in our ML only in the analysis using the no-admixture model. The other genetic clusters (Group 1 and 3) showed discordance between the two models and the ML topology (Fig. 3A). Lastly, discriminant analysis of the principal components (DAPC) of the e13_uSNPs matrix favored the presence of three clusters which agreed with those recovered in the structure analysis with the admixture model (Fig. S9).

### Testing for admixture—*E. titania*

Since our structure analyses showed evidence of admixture in *E. titania*, we ran TreeMix with four groups to identify patterns of migration. Our results consistently showed that when two migration events are considered, along with blocks of 10, 50, 100 and 1000 SNPs, one migration edge revealed gene flow from Group 4 to Group 1, with the topology in agreement with our ML analyses (Fig. 3B). When we deactivated the sample correction size on the 100 SNPs block, our results showed gene flow from Group 4 to Group 3. These migration edges showed low percentage ancestry received from Group 4 (Fig. 3B). However, TreeMix analysis without the sample correction size and 1000 SNPs block showed Group 2 as the ancestral population, and gene flow from Group 4 to Group 1 (figure not shown). Further, the diveRsity analysis revealed the highest relative migration between Group 4 towards Group 1, with lower migration between Group 4 towards Group 3 (Fig. 3C, Fig. S10). These results suggest highest migration from southern sites in the Sonoran desert north into the Mojave Desert (from Group 4 to Group 1), and a putative area prone to more gene flow. Lastly, our results suggested that Group 1 could be admixed between Group 3 and 4, with an unknown source yielding Group 2. Thus, we tested whether Group 1 and 2 (as a genetic cluster recovered by the structure analysis using the admixture model) are admixed between Group 3 and Group 4. Only one sample (*E. titania* | DMNS ZA.23689A, Fig. 3A) was most closely related to members of Group 3 and Group 4 than the members of Group 1 (Fig. 3D).

### Demographic history and species distribution modelling—*E. titania*

Our demographic history of *E. titania,* using Bayesian skyline plots analyzing 1000 and 2000 nucleotides, showed a general decrease in effective population size in the late Pleistocene, where the last glacial period peaked (~ 22,000 years ago), followed by a recent increase (Fig. 3E, Fig. S9). The SDM generated for *E. titania* based on current conditions indicate areas of suitable climate throughout low-elevations of the Mojave Desert and western Sonoran Desert (Fig. 3F). Areas with highest suitability occurred in the Colorado Desert, Death Valley and adjacent valleys, and the lower Colorado River Valley. The SDM predicted a much smaller distribution of suitable climates when hindcasted onto LGM conditions (Fig. 3F). Glacial climates were only predicted to be higher in the Colorado Desert, with moderate suitability in the Death Valley region, and a complete lack of suitable climate along the Colorado River Valley.

## Discussion

Our phylogenetic reconstructions using SNP data consistently support the monophyly of *Eremocosta*, in agreement with a previous 4-gene study and a recent morphological revision^7,8^. This, however, is where the similarities end. Our SNP-based analyses all indicate, with strong support, that *E. gigasella* specimens collected from canyonlands of the northern Chihuahuan Desert represent an undescribed species of *Eremocosta* that is sister to all other sampled species (Fig. 1B). Cushing et al.^7^ found strong support for a sister relationship between *E. gigasella* and *E. striata*. Likewise, our only sample of what we expect to be true *E. gigasella* (DMNS ZA.21950 in Fig. 1B, Fig. S1) grouped with *E. striata*, forming a long branch with an estimated late Miocene to Pliocene origin.

*Eremocosta striata*, *E. bajaensis*, *E. calexicensis*, and *E. titania* all formed monophyletic groups with strong support. This result was expected, and confirms that their traditional, morphology-based species descriptions represent real evolutionary entities. An unexpected result, however, was the position of *E. formidabilis* as sister to *E. titania*, despite a vast geographic gap between the two species’ distributions. *Eremocosta formidabilis* inhabits the southern Chihuahuan Desert, over 1000 km east of *E. titania* in the Mojave and western Sonoran deserts. We propose two scenarios that could explain this enormous disjunction.

First, the phylogenetic position of *E. formidabilis* could be incorrect due to contamination or missing data. The only available sample was collected in 2013 in Aguascalientes, México (the type locality of the species is Guanajuato, México). The specimen used was not necessarily preserved properly for DNA work. This could explain why we only obtained ~ 30% of the SNPs for this sample. That said, phylogenetic analyses with RADseq data have been demonstrated to perform well even with large amounts of missing data^13,14^. Furthermore, 13 of our other samples possessed as much or more missing data than *E. formidabilis* and were grouped with conspecifics with strong support. Another scenario is that the unexpected phylogenetic position of *E. formidabilis* is real. If this is the case, then *E. formidabilis* could be the result of a long-distance dispersal (LDD) event, as predicted to have occurred during the Pliocene in our ancestral area reconstruction. Additional samples would be needed to determine the cause of this curious result. In addition, México is undersampled for solifuges; therefore, there may be yet to be discovered diversity of the genus *Eremocosta* in the southern Chihuahuan desert region that may help to explain the position of *E. formidabilis*.

Morphological relationships among *Eremocosta* species, as assessed with our geometric morphometric analyses of chelicerae shapes (excluding the VDC, see “Methods”), highlight the difficulty in delimiting solifuge species without molecular data. By studying the evolution of shape as a continuous trait, multivariate analysis revealed a unique cheliceral shape morphology in males of *E. striata* and *E. titania*, and in females of *E. titania*. Strong sexual dimorphism in cheliceral morphology was only found in *E. titania*. All other chelicerae shape morphologies were remarkably conserved.

Despite the curious positions of the two abovementioned samples, ddRAD data allowed us to generate a robust phylogeny for *Eremocosta* with 100% bootstrap support values at all interspecific nodes (Fig. 1B), the first of its kind for any Solifugae genus. Given their difficulty to collect, most specimens were a decade old. Thus, our results corroborate those of other studies that underscore the power of genome-wide data for unlocking the genetic potential of museum specimens for molecular analyses^15–17^. Techniques like this are especially promising for taxa that are difficult to collect, like camel spiders.

Fossil records are sparse for Solifugae and nonexistent for Eremobatidae^7,18^. In spite of this limitation, our divergence dating analyses place the timing of diversification among *Eremocosta* spp. in a timeframe consistent with expectations given the histories of co-distributed taxa and the desert ecosystems they occupy. As in Cushing et al.^7^, initial (crown) diversification in *Eremocosta* was predicted to occur during the Miocene. Ancestral area reconstructions indicate that the genus probably colonized North American deserts from an ancestral region in the Sonoran Desert (Fig. 2, Fig. S5). However, given that some of the oldest lineages (*E.* aff. *gigasella*) occur in the Chihuahuan Desert, we suspect the genus actually diversified in an east-to-west pattern; moving from the Chihuahuan Desert, then Sonoran Desert, and on to the Mojave Desert, California Coastal Sage, and low to mid elevations of the Madrean Archipelago. Several animal groups are similarly distributed, but few share this east-to-west pattern. Among desert plants, however, phylogenomic evidence suggests that cactus genera *Cylindropuntia* and *Grusonia* originated in the Chihuahuan Desert during the mid to late Miocene region before migrating to and diversifying within other North American deserts^19^. Additionally, several phylogeographic studies have found that Chihuahuan Desert populations are sister to all other populations in deserts west of the Cochise Filter Barrier. Molecular clock-based analyses indicate that sister lineages found on either side of the barrier diverged at various times spanning the Miocene, Pliocene, and Pleistocene, best predicted by locomotive and thermoregulatory traits^11,20,21^. This timeframe corresponds with uplift of the Rocky Mountains and climatic differentiation between the Chihuahuan and Sonoran deserts. However, recent data from co-distributed snakes identified isolation by environment, rather than vicariance or dispersal, as the primary cause of divergence in the area^22^.

Interestingly, *E. calexicensis* and *E. striata* exhibit an east-to-west pattern as well. Although our sampling is sparse, a single *E. calexicensis* sample collected east of the Colorado River near Bullhead City, AZ is sister to all other samples to the west, suggesting a possible east-to-west colonization pattern across this ‘leaky’ river barrier^9^. Similarly, a single *E. striata* sample from Texas is sister to all other conspecific samples collected to the west. Molecular clock analyses suggest that this split is quite old, potentially dating to the Miocene, so we suspect that the Texas sample may represent a new *Eremocosta* species (Figs. 1, 2, Figs. S1–S5).

Diversification of the four most closely related species—*E. striata*, *E. bajaensis*, *E. formidabilis*, and *E. calexicensis*—was estimated to occur during the late Miocene to Pliocene (Fig. 2). Of these, *E. bajaensis* is the oldest, with an estimated divergence time of about 7–5 Ma when using the arthropod rate calibration. This timeframe overlaps the time when a flooding event formed the northern third of the Gulf of California, reaching as far north as San Gorgonio Pass. Fossil data indicate that the northern gulf was flooded near synchronously at 6.3 ± 0.1 Ma^10^. Marine waters extending north through the Salton Trough would have effectively isolated *Eremocosta* inhabiting the Peninsular Range. If true, then the general arthropod rate has proven to work remarkably well with camel spiders, and vicariance caused by sudden flooding of the northern Gulf might be useful for calibrating molecular clocks in studies of other taxa inhabiting the region.

By integrating phylogenetics, structure and DAPC analyses, and species distribution modelling, we were able to characterize fine-scale genetic patterns in *E. titania*, a first for camel spiders. Results indicate that the species comprises four geographically structured groups; two in basins along the western fringe of the Sonoran Desert (Anza-Borrego Desert and Coachella Valley), one in the Mojave and Sonoran ecotone (near Twentynine Palms), and another found throughout the western Mojave Desert (Fig. S10). All except for the Mojave group were narrowly distributed in desert valleys. The Mojave group was much more widely distributed, ranging from the western Mojave Desert in California and northeast into southern Nevada. The group probably occurs throughout low elevations of the Mojave Desert, as predicted by our species distribution model (Fig. 3F).

The distribution of *E. titania*, especially the Mojave group, could have been reduced during the last glacial maximum, restricted to low-elevation areas in the western Sonoran Desert where the three narrowly distributed groups occur (Fig. 3F). Distribution modelling of other arthropods have identified the same general area as a desert refugium as well^23,24^. Therefore, we suspect that the four groups diverged when they became repeatedly isolated in a western Sonoran refugium during Pleistocene glacial cycles. The LGM model predicts that climates were not suitable at all Mojave group sites, so the Mojave group’s current distribution is likely a product of significant post-glacial range expansion. This interpretation is supported by results from the demographic analyses of SNP data, which depicts late Pleistocene growth in effective population size for *E. titania* (Fig. 3E).

Although the largest swath of suitable late glacial habitat occurs in the south, the LGM model predicts that Death Valley could have also been a desert refugium for the Mojave group. The valley was flooded during much of the Pleistocene, forming Lake Manly, but suitable habitat could have been available for *E. titania* along the shoreline and adjacent areas higher elevations. Arachnids are known to exhibit phylogeographic patterns consistent with a model of leading-edge colonization^14,24^, so if Death Valley was a refugium, then we should see a pattern of decreasing genetic diversity with distance from the valley. Sample sizes were not large enough to address this question using population genetics, but individual heterozygosity values for Mojave group individuals were greatest at middle latitudes (Fig. S12). Thus, *E. titania* may have expanded from two glacial refugia, one in Death Valley and another at the southern end of the range. Additional sampling, especially in Death Valley, would be needed to address this hypothesis.

Migrate analyses provide an interesting picture of varying levels of gene flow among the four *E. titania* groups (Fig. 3B–D). Unsurprisingly, the southernmost groups in the western Sonoran Desert (Groups 2–4) exhibits about equal and moderate levels of gene flow between them. The strongest signal of gene flow, however, comes from the southernmost group (Group 4) north to the Mojave group (Group 1), with very little movement of genes in the other direction. This result may at first seem unlikely given that Groups 2 and 3 are more geographically proximate to the Mojave group. Additionally, desert habitat in the area is divided by both the easternmost extension of the Transverse Ranges (Little San Bernardino Mts) and northernmost Peninsular Ranges (San Jacinto Mts). However, given the difficulty in collecting camel spiders, our sampling of *E. titania* in the western Sonoran Desert was limited, and did not include known populations that occur further east in the Salton Trough. These eastern populations may have a less impeded connection with Mojave group samples to the north, thus permitting gene flow to bypass the other groups and mountain ranges that bisect them.

Ultimately, the majority of phylogeographic structure within *E. titania* occurs in the western Sonoran Desert. This region, also known as the Colorado Desert, has been demonstrated to harbor significant genetic structuring in other desert animals as well; i.e. sidewinders^25,26^, toads^27^, night lizards^28^, pocket mice^29^, and scorpions^30^. As such, the area has been identified as a hotspot for genetic diversity^31^. *Hadrurus arizonensis*, which are large, arid-adapted scorpions, exhibit a similar pattern of genetic differentiation in low elevation refugia and subsequent expansion throughout the Mojave Desert^24^. Conversely, a lack of significant genetic differentiation was observed in flat-tailed horned lizard (*Phrynosoma mcallii*) populations.

Taken together, genome-wide SNP data and species distribution modelling provide compelling evidence that *E. titania* was severely impacted by pulses of cooler and wetter climates associated with Pleistocene glacial cycles. These large, arid-adapted predators were probably once restricted to isolated low-elevation refugia where climates remained xeric during glacial periods. As climates warmed, the species then successfully colonized new areas of suitable habitat as woodlands were predominately replaced by desert scrub ecosystems throughout the Mojave Desert.

## Methods

### Taxon sampling, RAD sequencing, and assembly

Genomic DNA was extracted from 68 museum preserved specimens as well from material collected between 2017 and 2018 (Table S2). All specimens used were from the DMNS arachnology collection and species identifications were verified by at least two experts. Appropriate permissions from museum authorities at DMNS were obtained for using material from the museum in this study. Data from all specimens used can be accessed via the Symbiota Collections of Arthropods Network (https://scan-bugs.org/portal/index.php). Sixty-five of the samples represented six of the seven species in *Eremocosta* (only *E. gigas* is missing), and three were outgroups; two samples of *Hemerotrecha branchi* (Eremobatidae) and one *Ammotrechula* sp. (Ammotrechidae). Library preparation and sequencing followed our recent protocols^14,32^. In brief, we used two restriction enzymes (EcoRI-HF and ClaI) to make cuts for adapter ligations and MspI for dimer cleaving (all enzymes from New England Biolabs, Ipswich, MA). All samples were pooled and subjected to 2 × 150 paired-end sequencing on a full lane of an Illumina HiSeq X at Admera Health (South Plainfiled, NJ). Raw reads were demultiplexed and assembled using iPyRAD v. 0.9^33^ with default parameters. Different alignments were created by requiring loci to be shared by at least 17, 22, and 33 taxa. The amount of missing data was analyzed and samples with more than 95% of missing data were dropped by repeating the assembly. We created new alignments that required loci to be shared by at least 21 taxa (hereafter referred to as alignment ‘m21’). Assembly statistics are reported in Table S3.

### Phylogeny, divergence dating, and ancestral area reconstruction

We used the concatenated matrices of SNPs (m21_SNPs) and uSNPs (m21_uSNPs) to infer phylogenetic relationships among *Eremocosta* species. For each of these matrices, we conducted maximum likelihood (ML) analyses using IQ-TREE v. 1.6.6^34^ implementing ModelFinder^35^ and ultrafast bootstrap resampling^36,37^.

Our team’s previously published eremobatid chronogram based on four genes (COI, 16S, H3, and 28S) suggests that *Eremocosta* species shared a common ancestor during the Miocene, between 10 and 18 Ma^7^. This estimation was calculated using fossil calibrations for outgroup lineages, as well as a uniform prior placed on a node shared by sister species found on each side of the Trans-Mexican Volcanic Belt. However, the substitution rates derived from this approach were high. For example, a rate of 0.0379 substitutions/site per million years was estimated for COI, which is more than twice as fast as rates estimated for spiders^38^ and scorpions^30^. Therefore, we also reanalyzed the original four-gene dataset in BEAST v 1.10^39^ without the fossil and biogeographic calibrations, instead using a rate calibration commonly used for COI in arthropods (0.0169 subs/site/my^40^). All other parameters were set as in the previous analysis: unlinked substitution and clock models across the four partitions, a strict clock (ucld.stdev values were less than 1.0 in preliminary runs with relaxed clocks), Yule speciation process, and four mcmc runs for 50 million generation each, sampling every 5000.

We then used divergence date estimates from the original chronogram as well as the new arthropod rate-based chronogram to calibrate two different molecular clocks for *Eremocosta* with our RAD data. Specially, we used the putative origin of Eremobatidae (where *Eremocosta* split from *Hemerotrecha*), and the divergence of *Eremocosta* as recovered in (a) Cushing et al.^7^ and (b) our new analysis. Divergence dates were estimated by analyzing m21 using the approximate likelihood calculation^41^ as implemented in *baseml* and *mcmctree*, both part of the PAML v. 4.9 software package^42^. The ML tree inferred from m21_SNPs was used as the input tree calibrated using the putative origin of Eremobatidae and the divergence of *Eremocosta* as previously discussed. Four Bayesian inference chains were run for 10 million post-burnin generations (burn-in of 10,000), and an independent model rate of evolution; convergence of chains was confirmed using MCMCTreeR^43^.

We constructed a species distribution matrix to estimate ancestral areas for *Eremocosta* lineages by designating each terminal taxon to the ecoregions^44^ they inhabit (Table S4) with the RASP v. 4.2 package^45^. We fitted the data to six models as implemented in the R package BioGeoBEARS^46^: DEC, DEC + j, DIVALIKE, DIVALIKE + j, BAYAREALIKE and BAYAREALIKE + j. Following Turk et al.^47^, we omitted the outgroups as well as our single sample of *E. formidabilis* due to the possibility of contamination or bias from missing data (see “Discussion”). We ran all models in RASP with a maximum number of areas occupied set to two. We then compared all six models using the Akaike information criterion (AIC) values and Akaike weights (AICw). The model DEC + j was favored (Table S5).

### Evolution of sexual dimorphism

*Eremocosta* morphologies are largely conserved with most species-level differences occurring in male chelicerae. Therefore, we explored sexual dimorphism in cheliceral morphology within a phylogenetic context to determine if species diverged morphologically as they colonized and adapted to new areas, as predicted by ancestral area reconstructions (see section above). Cheliceral shape variation was characterized using the geometric morphometric technique of elliptic Fourier analysis (EFA) with the R package Momocs^48^, following previous studies^49^. We used Adobe Photoshop^®^ to outline monochromatic versions of cheliceral photographs that were published in a revision of the genus^8^. Outlines were imported into R, converted into lists of coordinates, and aligned using the *calibrate_harmonicpower* function in Momocs. Additional arguments for the EFA included the normalization of coefficients, and a single smoothing iteration. Resulting coefficients were summarized using a Principal Component Analysis (PCA) with the principal components (PCs) used to visualize the variation of the cheliceral shape in the morphospace.

We used a MANOVA to compare the shapes between sexes after the EFA and PCA. Only species for which both female and male photographs were available were included. Deformations between the shapes of both sexes were determined using Thin Plate Splines with the tps_iso function in Momocs. Euclidean distances were calculated between females and males using the truss function with the scores of the first PC. This resultant Euclidean distance represents the degree of sexual dimorphism, which was plotted onto our dated topology as a function of the variation of cheliceral dimorphism through time. This approach explores the general morphology of chelicerae and known differences between ventro-distal concavity (VDC) should not significantly influence the results.

### Population structure—*E. titania*

To determine if Pleistocene climate fluctuations impacted *Eremocosta*, we conducted a phylogeographic analysis of *E. titania*, the species for which we had the most samples. First, we generated an assembly with loci shared by at least 13 of the 17 *E. titania* samples (‘e13’—Assembly statistics are reported in Table S2). Using this assembly, we assessed population structure by using the Bayesian MCMC clustering method implemented in Structure v. 2.3.4^50^, with the e13 unlinked SNPs matrix (e13_uSNPs). Correlated allele frequencies without using prior population information and the admixture and no-admixture models were implemented for 10 independent runs for k values (2–6) with 10,000 mcmc cycles, with a burn-in of 1000 iterations. The best-fit K value was determined using the log probabilities of X|K^50^ and the Delta K method^51^ as implemented in the online software Structure Harvester v. 0.6.94^52^. The multiple runs of the selected K values were aligned using CLUMPP v. 1.1.2^53^ with the greedy algorithm. Additionally, we conducted a Discriminant Analysis of Principal Components (DAPC) using the e13_uSNPs dataset and the package ADEGENET^54^ in R 3.5.2. The value of clusters (k) was constrained to “3” as suggested by BIC values using the first 13 PCs, retaining three axes in Discriminant analysis (DA).

### Testing for admixture—*E. titania*

Next, we determined the number of putative admixture and migration events in the resulting populations from the structure analysis within *E. titania* using Treemix v. 1.13^55^. For this analysis, we used the allele frequencies from assembly ‘ec13’, our selection of at least two “migration” events (option -m 2), with or without the sample size correction (-noss), and blocks of 10, 50, 100, and 1000 SNPs to account for linkage disequilibrium. The tree was unrooted. In addition, we calculated the relative migration rates among groups with *divMigrate* from the diveRsity package^56^ using Jost’s D and Nei’s Gst.

Similarly, the three-population tests (*f statistics*) measure allele frequency correlations between populations as first introduced in Patterson et al.^57^. These statistics are used to test for admixture in a target population from two source populations, or to measure the shared genetic drift between two populations, rooted with an outgroup. Based on our results from the Treemix analysis, we sought to determine if groups 1 and 2 were the result of an admixture event between groups 2 and 3.

### Demographic history—*E. titania*

We reconstructed the demographic history of *E. titania* using the multi-locus method Extended Bayesian Skyline Plot (EBSP) as implemented in BEAST v 2.5.2^58^. As input data, we randomly selected different compositions of sequences from our e13_uSNP matrix (50, 100, 1000 and 2000 nucleotides). The HKY substitution model was implemented with a strict clock model with default parameters (since no mutation rate is known for Solifugae genomes). The chain length was set up to 100 million generations sampling every 5000 states implemented in two independent runs.

### Species distribution modelling—*E. titania*

We developed species distribution models (SDMs) for *E. titania* using coordinates for the 17 sites where our samples were collected. We chose to use only samples for which we had genetic data confirming their identity. SDMs were constructed using bioclimatic data representing current (1950–2000) and last glacial maximum (LGM) Bioclimatic interpolations downloaded from the WorldClim database^59^ at 2.5′ (ca 4 × 4 km) resolution. We clipped the layers an extent bounding the known range of *E. titania*, as potentially accessible desert habitats in adjacent areas (30.0–39.0° N and 111.0–120.0° W). We screened all 19 bioclimatic layers in each data set for multicollinearity using ENMTools 1.3^60^ and removed highly correlated (Pearson’s *r*^2^ > 0.9) variables. For highly correlated pairs, we retained the layer that contributed the most in preliminary runs using all 19 layers. This approach yielded the following final predictor layers: Bioclim 1, 2, 3, 4, 5, 8, 9, 13, 14, 15, and 18.

We used Maxent 3.4.1^61^ to construct a present-day SDM, and then projected the model onto the paleo climatic conditions estimated for the LGM. We ran five replicates using cross-validation (equivalent to 20% testing), complementary log–log (cloglog) transformation^62^, the maximum number of iterations set to 10,000, a random seed, and application of the fade by clamping. We optimized the regularization multiplier by using ENMTools to select the best model based on the corrected Akaike Information Criterion (AIC_c_) scores among models constructed using beta regularization multipliers of 1–10. The default multiplier (1) was considered optimal, and we used default settings for all remaining parameters.

We used ArcGIS 10.1 (ESRI, Redlands, CA, USA) to visualize the distribution of climates suitable for *E. titania* by using a color ramp for values above the “minimum training presence” threshold. This threshold is appropriate because it sets the omission rate to zero, and none of our samples should be omitted because coordinates were collected in the field (not georeferenced).

## Supplementary Information

Supplementary Information.
