# Supplementary material for: Diversification and post-glacial range expansion of giant North American camel spiders in genus Eremocosta (Solifugae: Eremobatidae)
Source: Sci Rep. 2021 Nov 11;11:22093. doi: 10.1038/s41598-021-01555-1 (PMC8586242; doi:10.1038/s41598-021-01555-1)
Supplement: Supplementary file 1 — Supplementary Information. [file 41598_2021_1555_MOESM1_ESM.pdf]

Supplementary Material for

Diversification and post-glacial range expansion in giant North American camel spiders (Solifugae: Eremobatidae: Eremocosta)

Carlos E. Santibáñez-López, Paula E. Cushing, Alexsis M. Powell, Matthew R. Graham

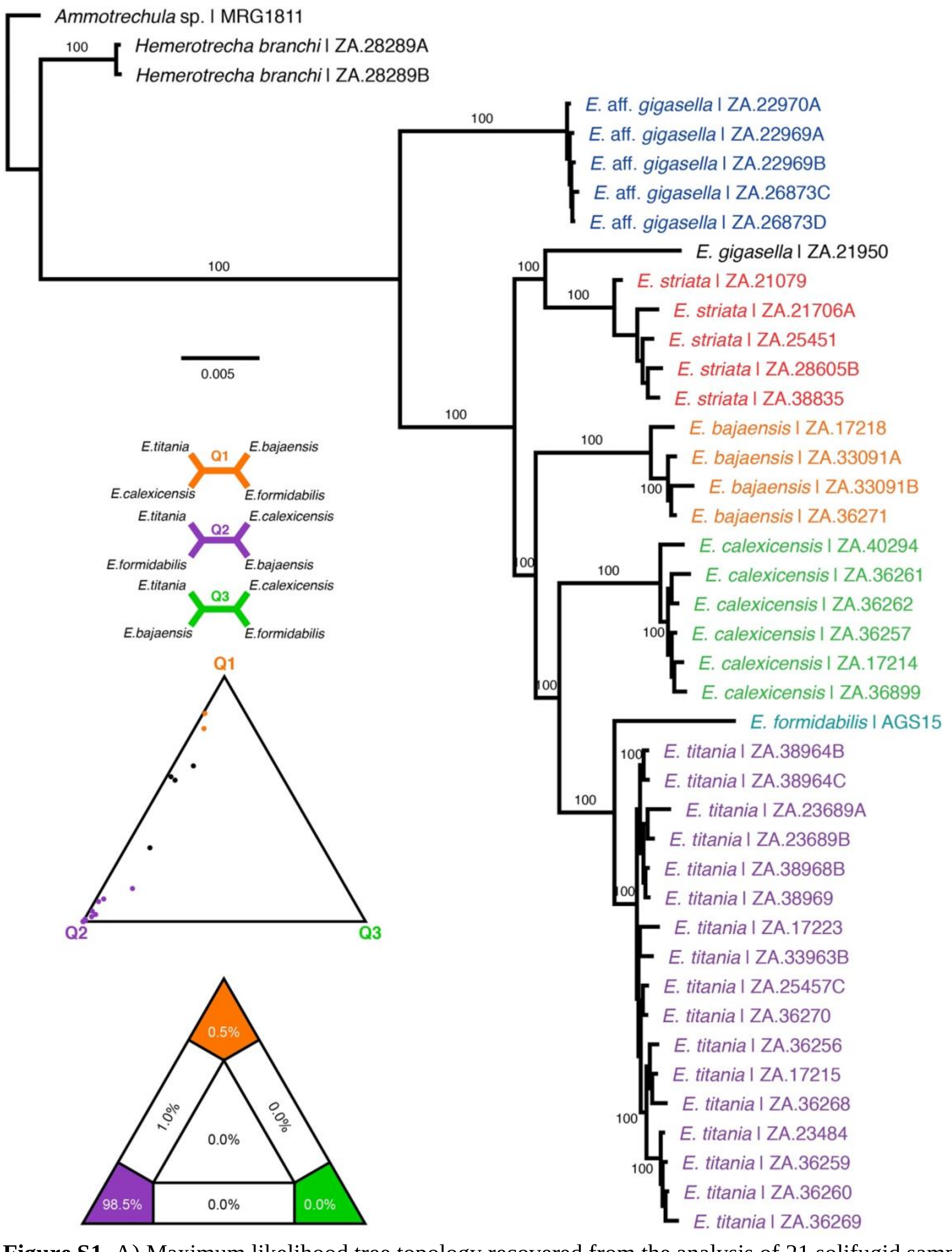

Figure S1. A) Maximum likelihood tree topology recovered from the analysis of 21 solifugid samples and 521,343 sites using the GTR+F+R2 model as selected by ModelFinder (m21; lnL=-908151.1928). Numbers on nodes indicate ultrabootstrap support. Nodes without numbers were supported by less than 100%. B-D) Quartet likelihood mapping (QLM) of three alternative quartet topologies (B) to test the position of *E. titania* and their respective percentage of the informative regions of the map (C-D).

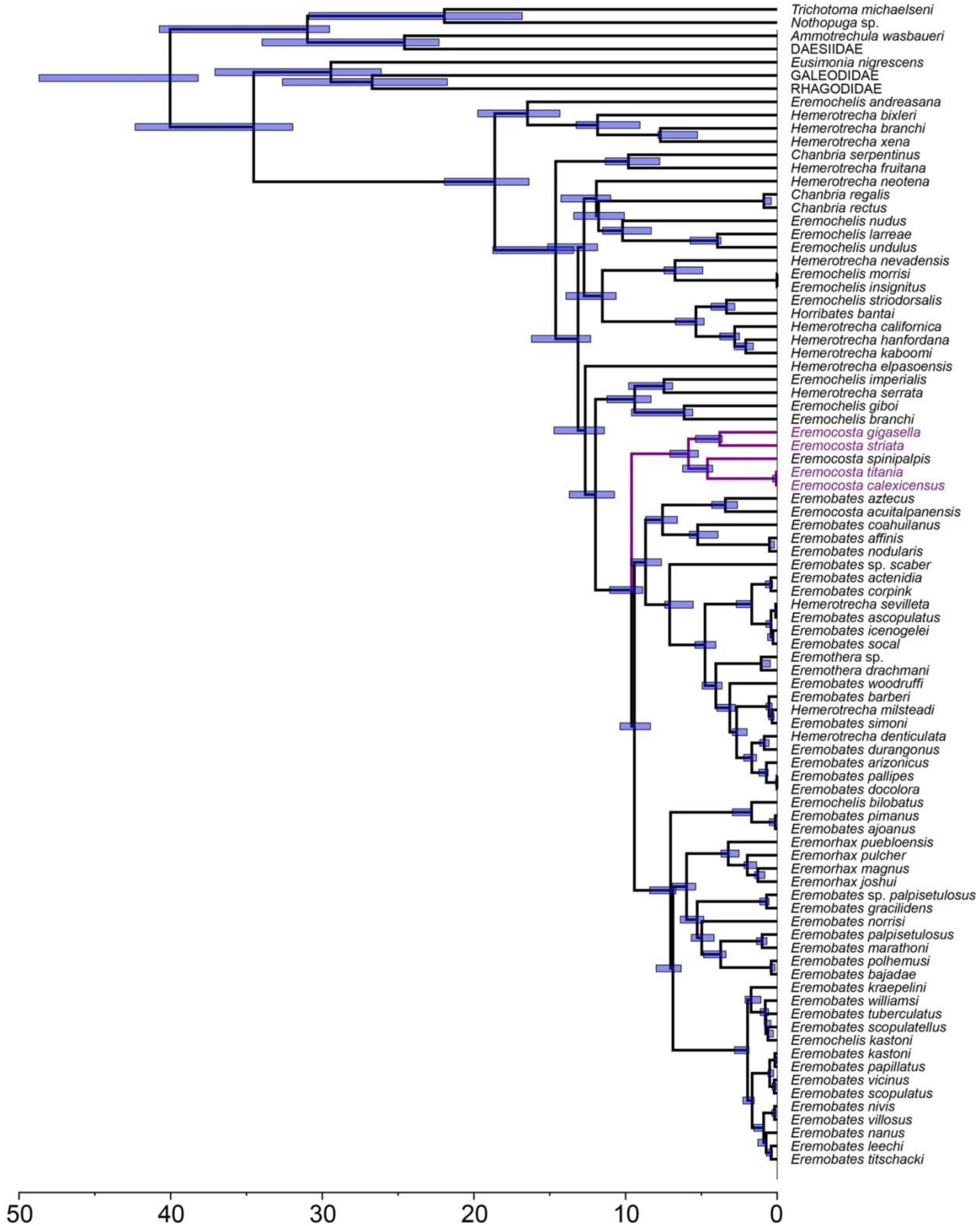

Figure S2. Rate-calibrated chronogram estimated by BEAST using Cushing et al. (2015) multilocus dataset, calibrated with the general arthropod rate (0.0169; CITE). Bars represent highest posterior densities (95%) around mean date estimates. Genus *Eremocosta* is highlighted in purple.

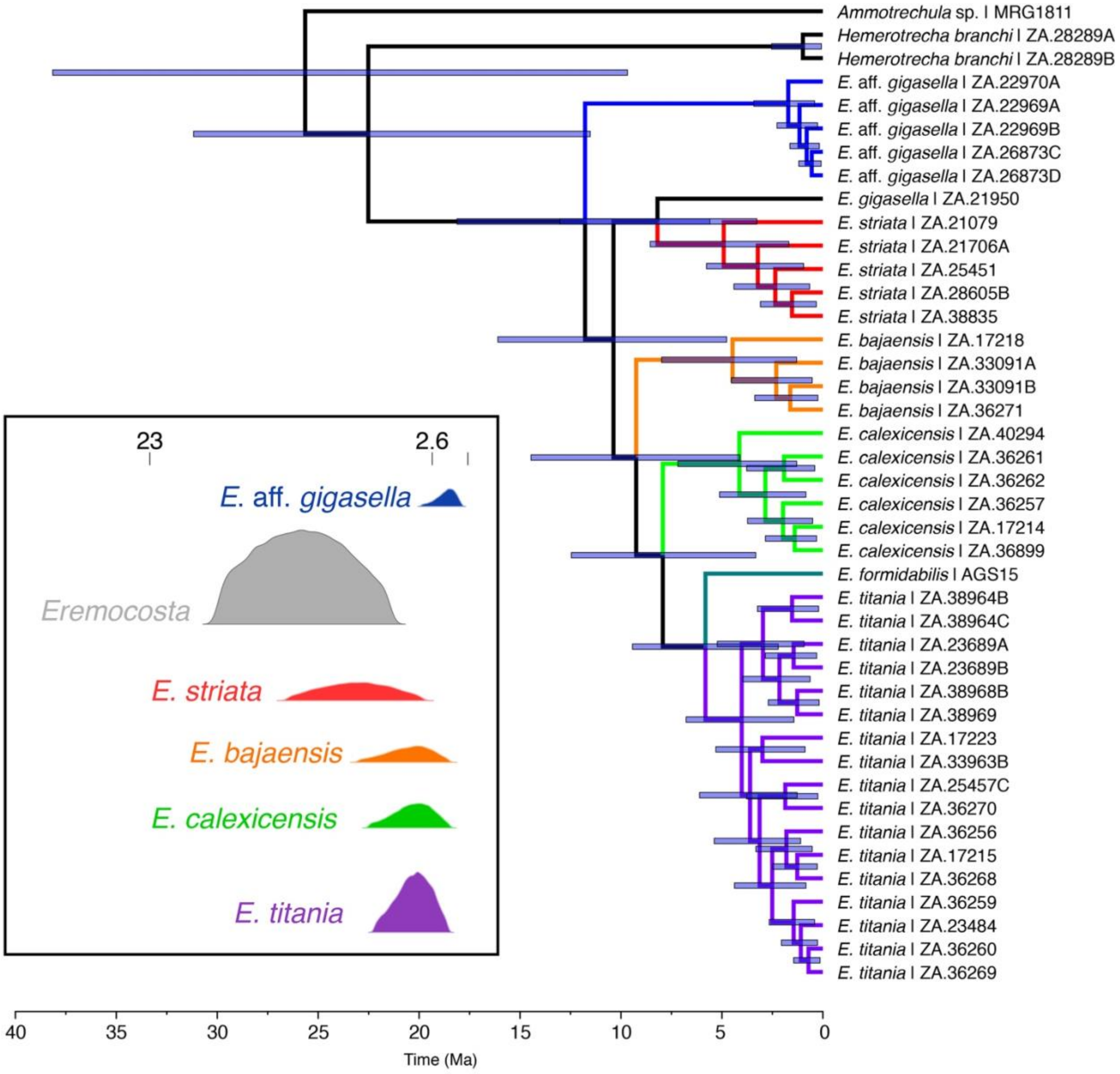

Figure S3. Chronogram of *Eremocosta* species derived from Maximum-likelihood analysis of 33,392 loci, using independent rates as a clock model generated by mcmctree. Node ages were computed in a time-calibrated analysis using the stem age of *Eremocosta* as recovered by Cushing et al. 2015. Blue bars depict 95% credibility intervals of node ages.

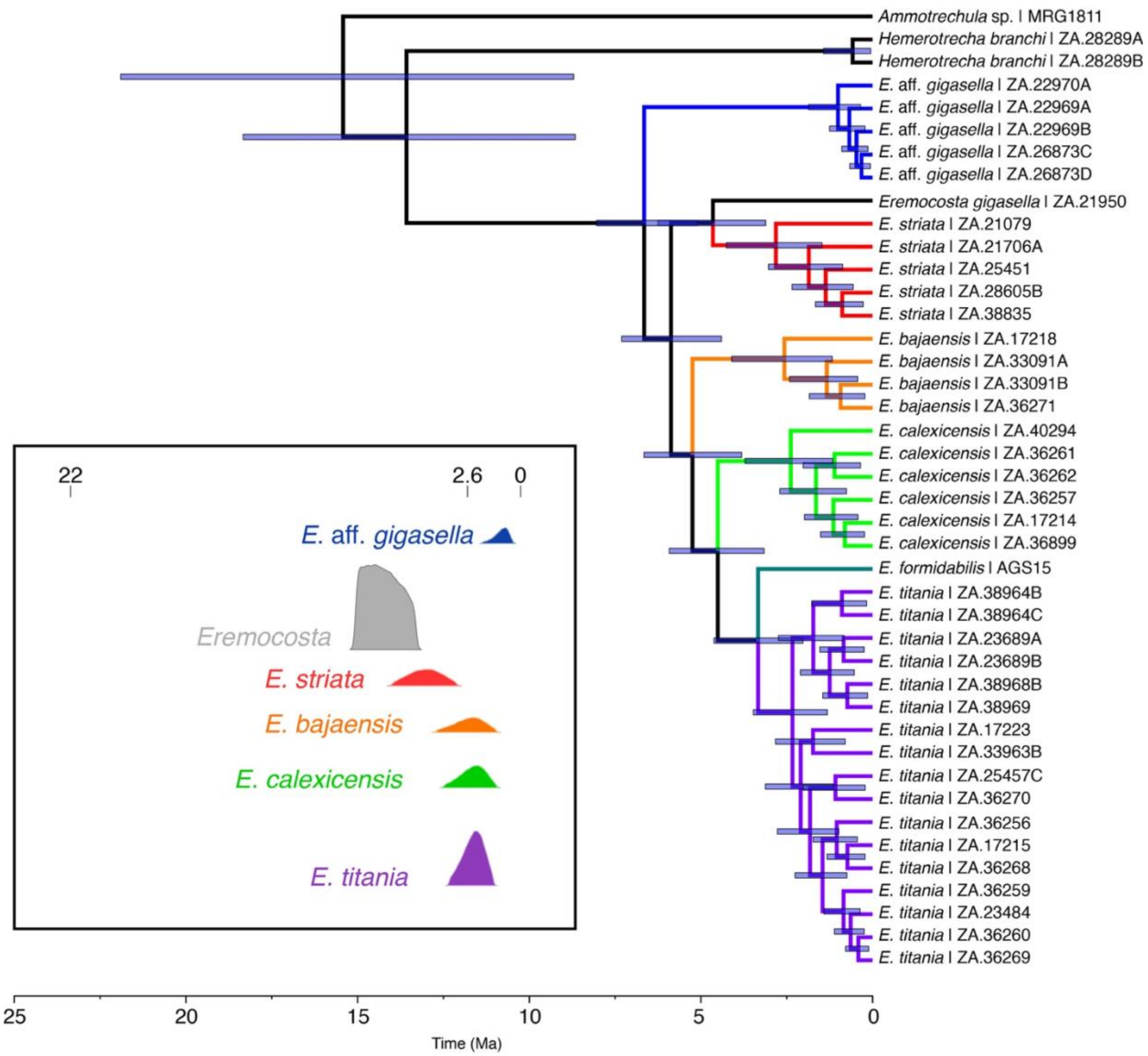

Figure S4. Chronogram of *Eremocosta* species derived from Maximum-likelihood analysis of 33,392 loci, using independent rates as a clock model generated by mcmctree. Node ages were computed in a time-calibrated analysis a mutation rate commonly used for arthropods (Papadopoulou et al., 2010). Blue bars depict 95% credibility intervals of node ages.

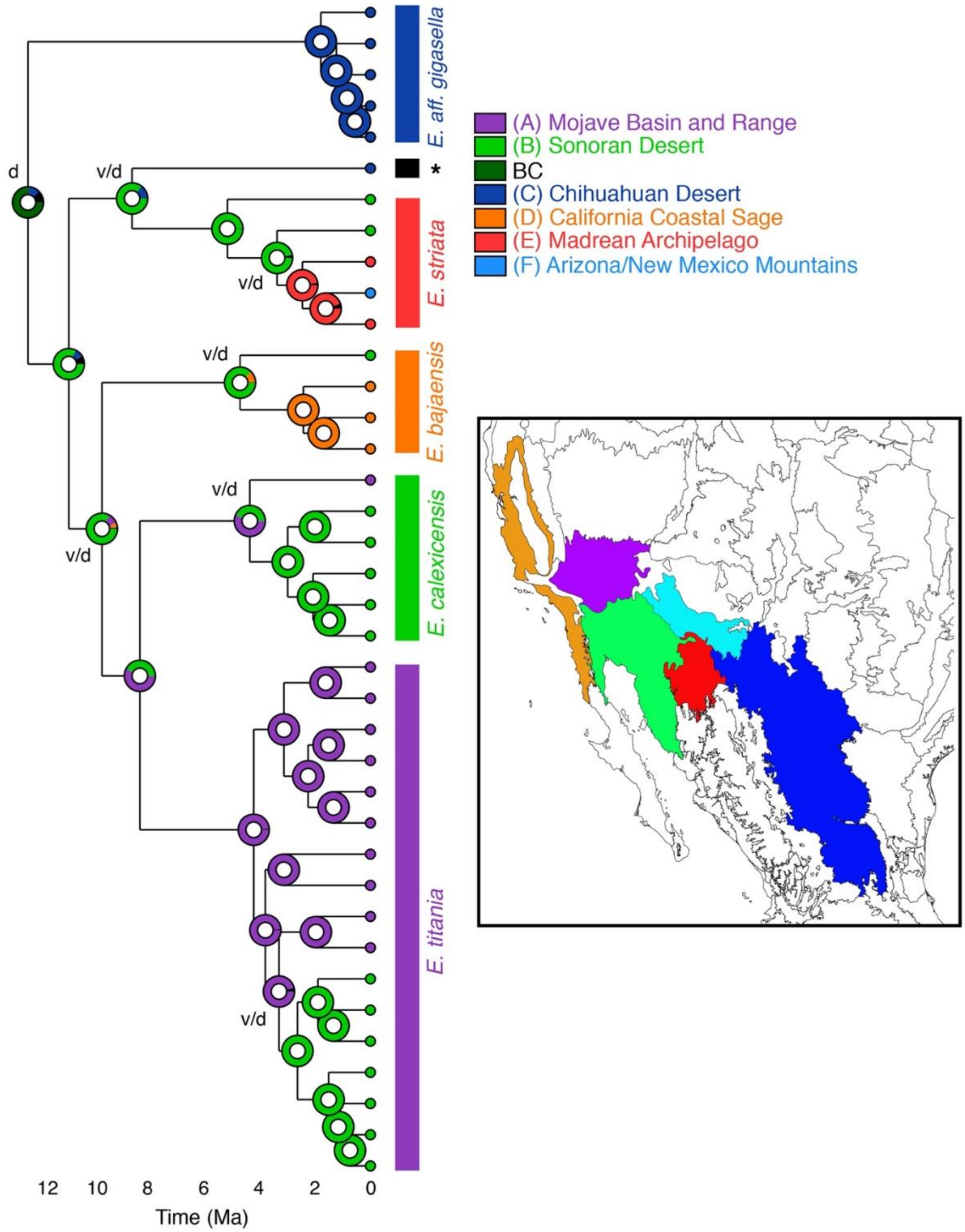

Figure S5. Ancestral areas estimated by RASP using *DEC+J* and a maximum of two areas were mapped on MRCA nodes for each species in our favored time-calibrated topology (Figure S4). Ecoregion map was generated in ArcGIS using a base map (Ecoregion level III) from the United States Environmental Protection Agency (EPA) [Available at: epa.gov/eco-research/ecoregions-north-america].

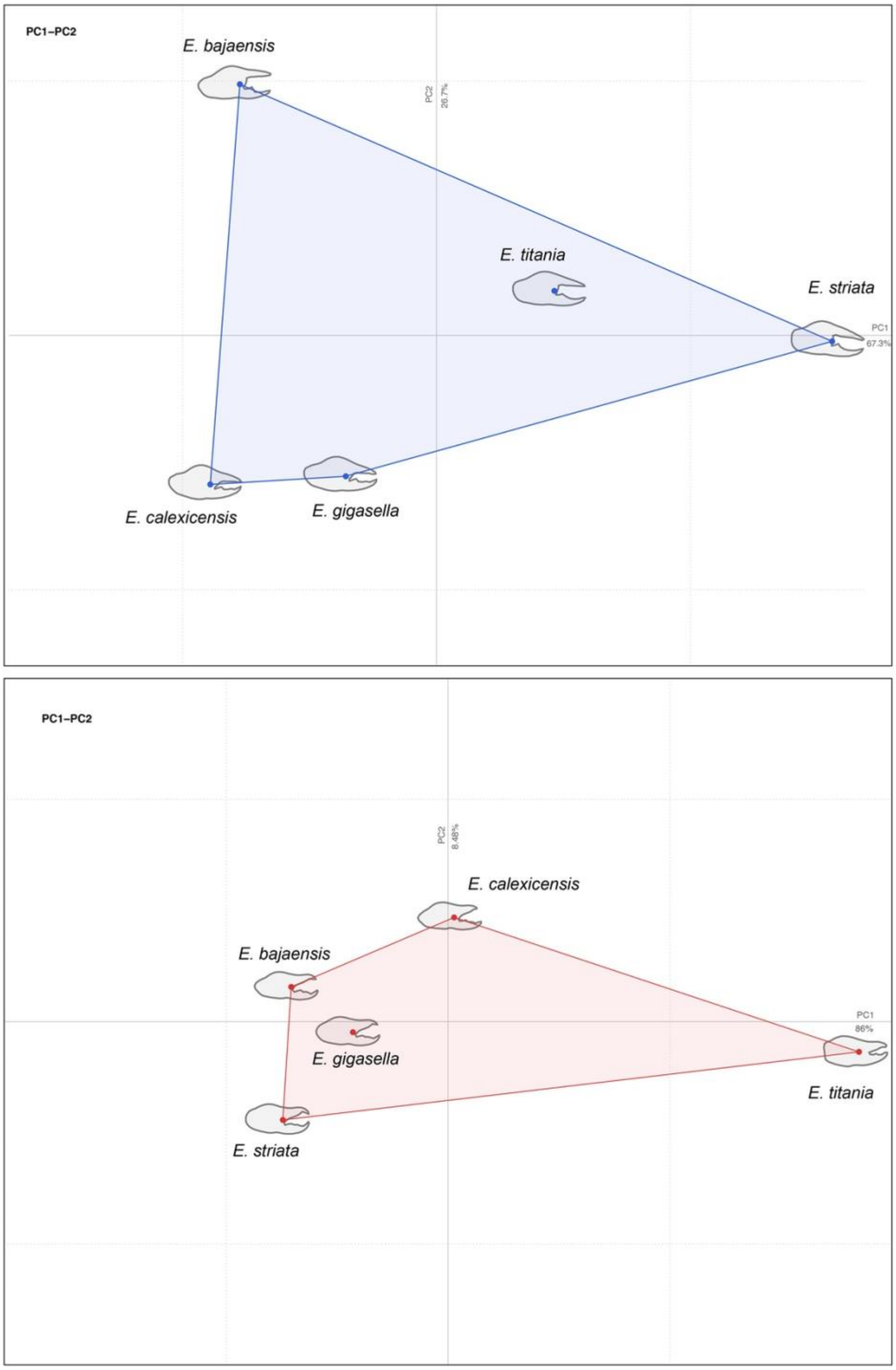

Figure S6. Visualisation of the chelicerae morphospaces showing the variation in the principal components 1 and 2 within *Eremocosta* males (top, in blue) and females (bottom, in red). Drawings correspond to the silhouettes recovered by *momocs*.

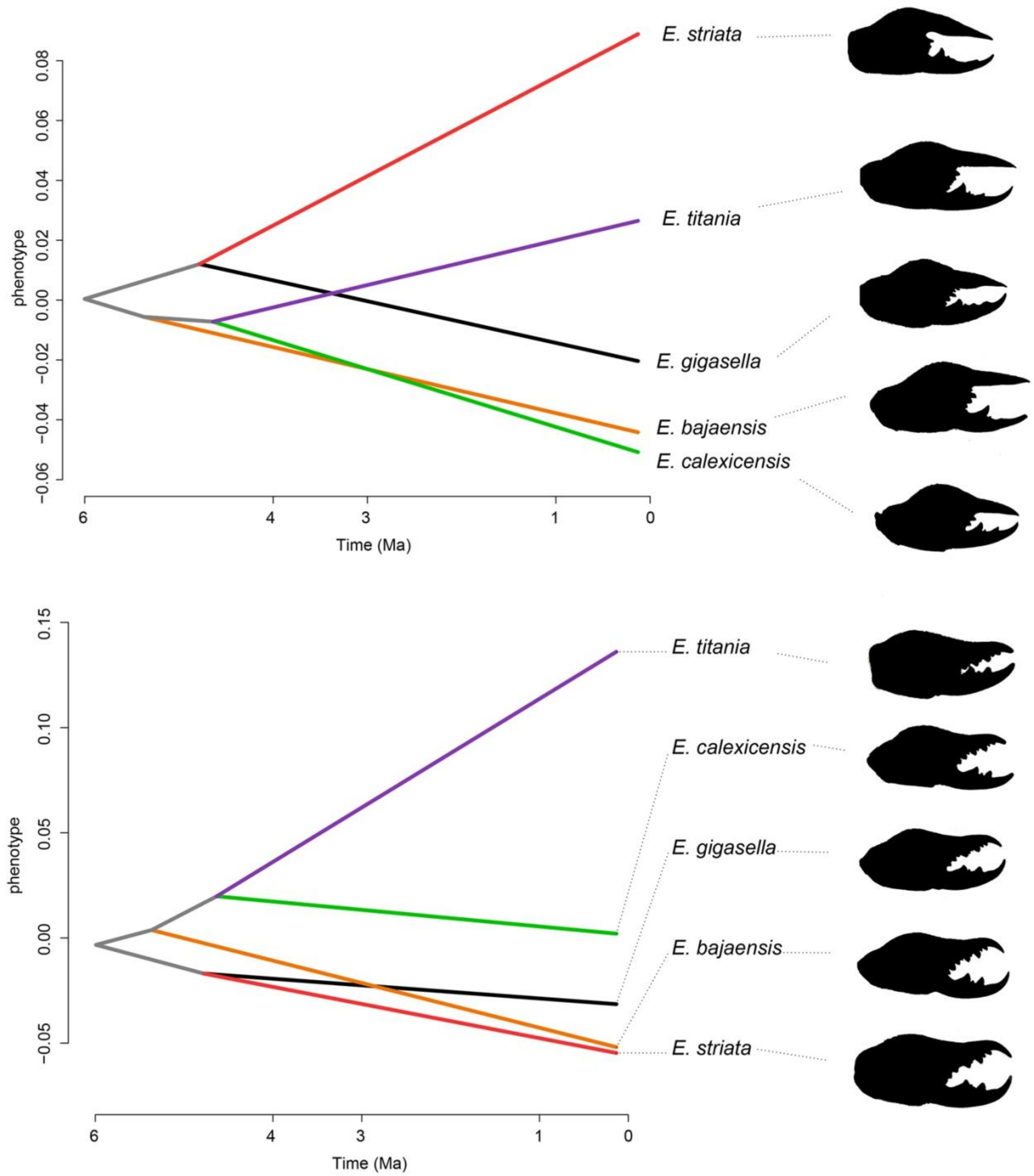

Figure S7. Visualization of the principal component values of the chelicerae morphospaces of females (top) and males (bottom), as a function of phylogenetic relationships recovered from the dated molecular tree from mcmctree. Silhouettes were drawn with Adobe Photoshop© using as base the photographs in Cushing et al. (2018) [See main text].

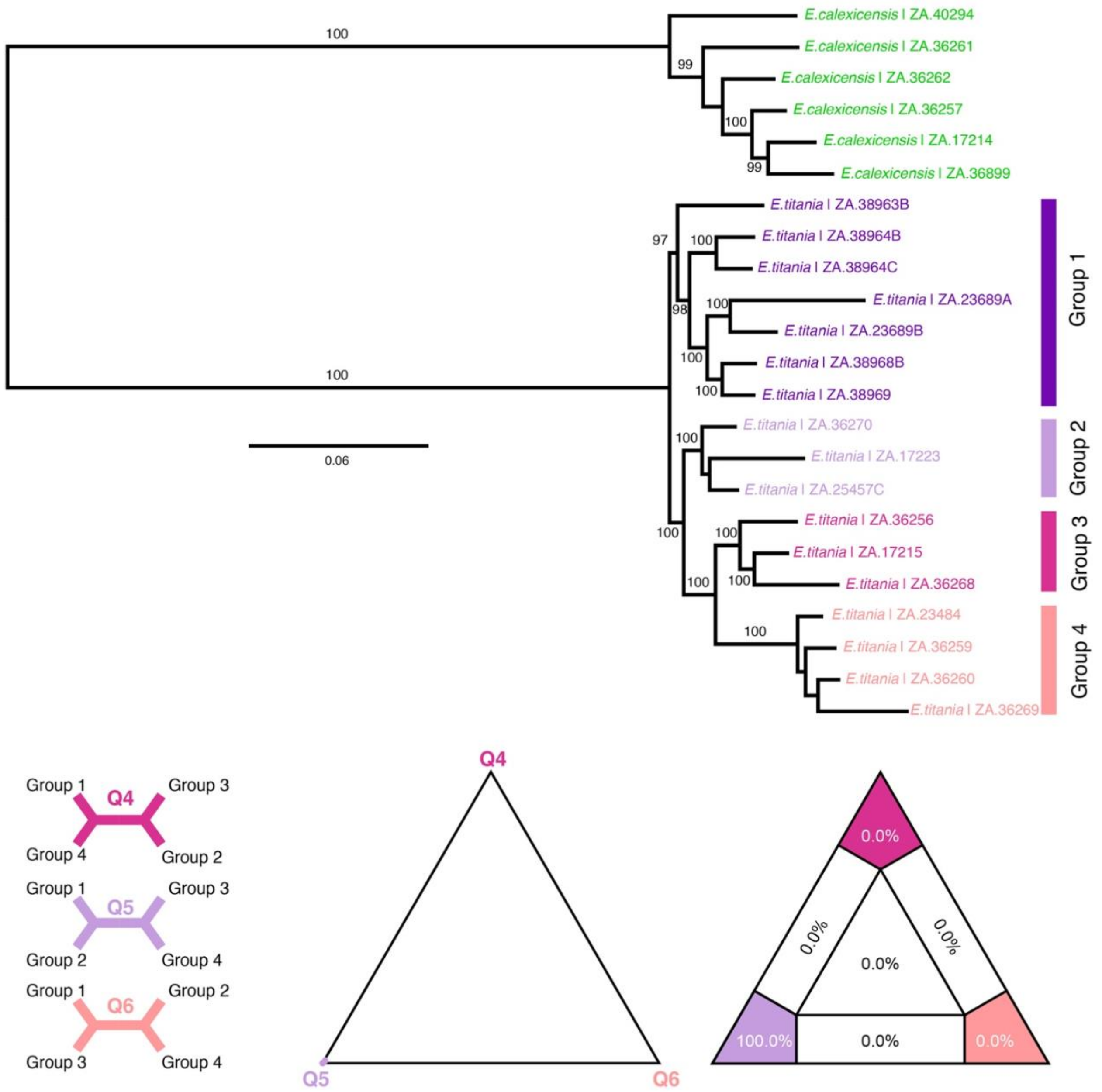

Figure S8. A) Maximum likelihood tree topology recovered from the analysis of 17 samples of *E. titania* and six samples of *E. calexicensis*, and 65,674 nucleotide sites using the GTR+F+R2 model as selected by ModelFinder (Ln= -292,482.2174). Nodes without numbers were supported by less than 95%. B-D) Quartet likelihood mapping (QLM) of three alternative quartet topologies (B) to test the position of the *E. titania* population 4 and their respective percentage of the informative regions of the map (C-D).

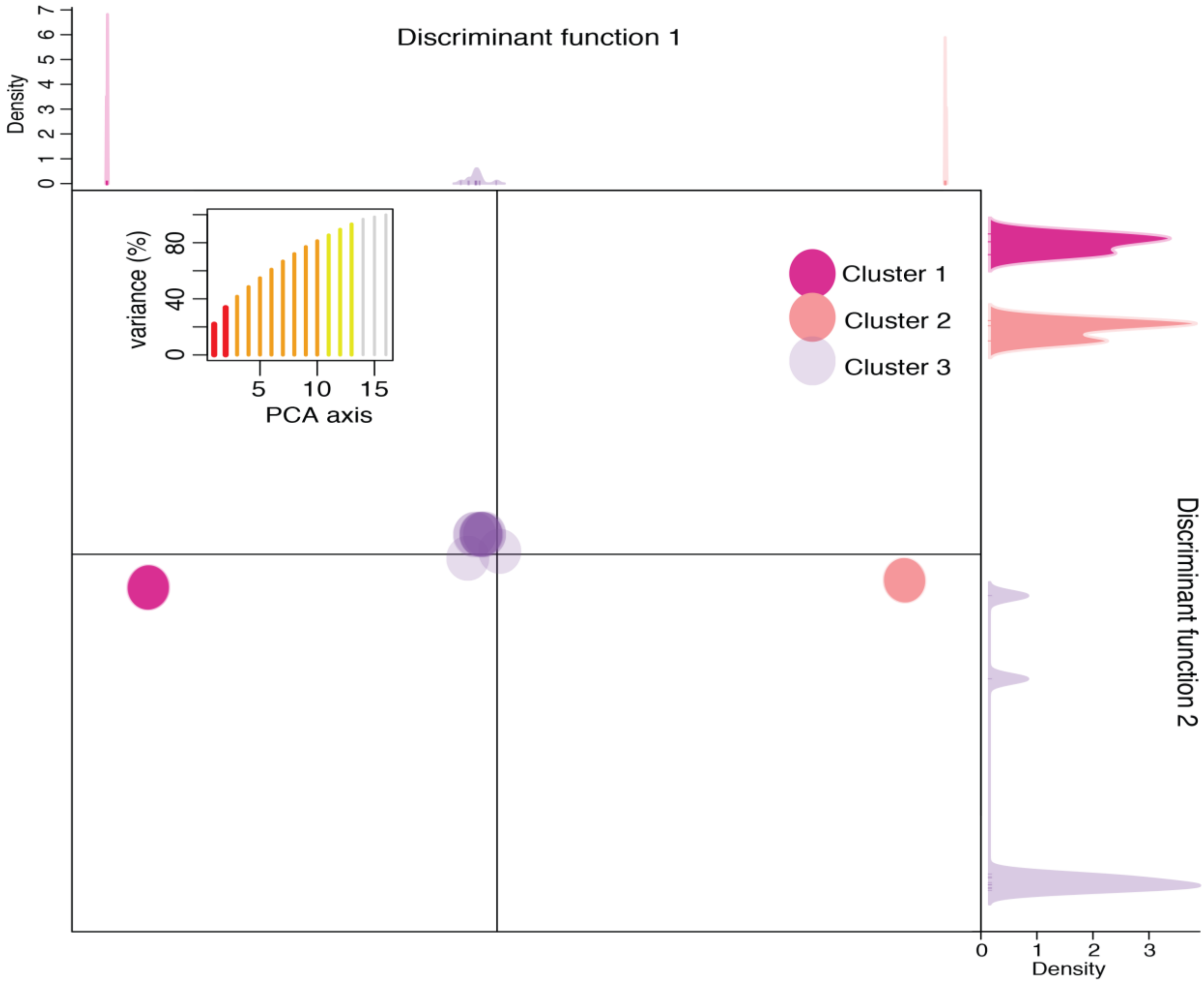

Figure S9. Visualization of the discriminant function (DAPC) using unliked SNPs from 17 samples of E. titania showing three distinct genetic clusters.

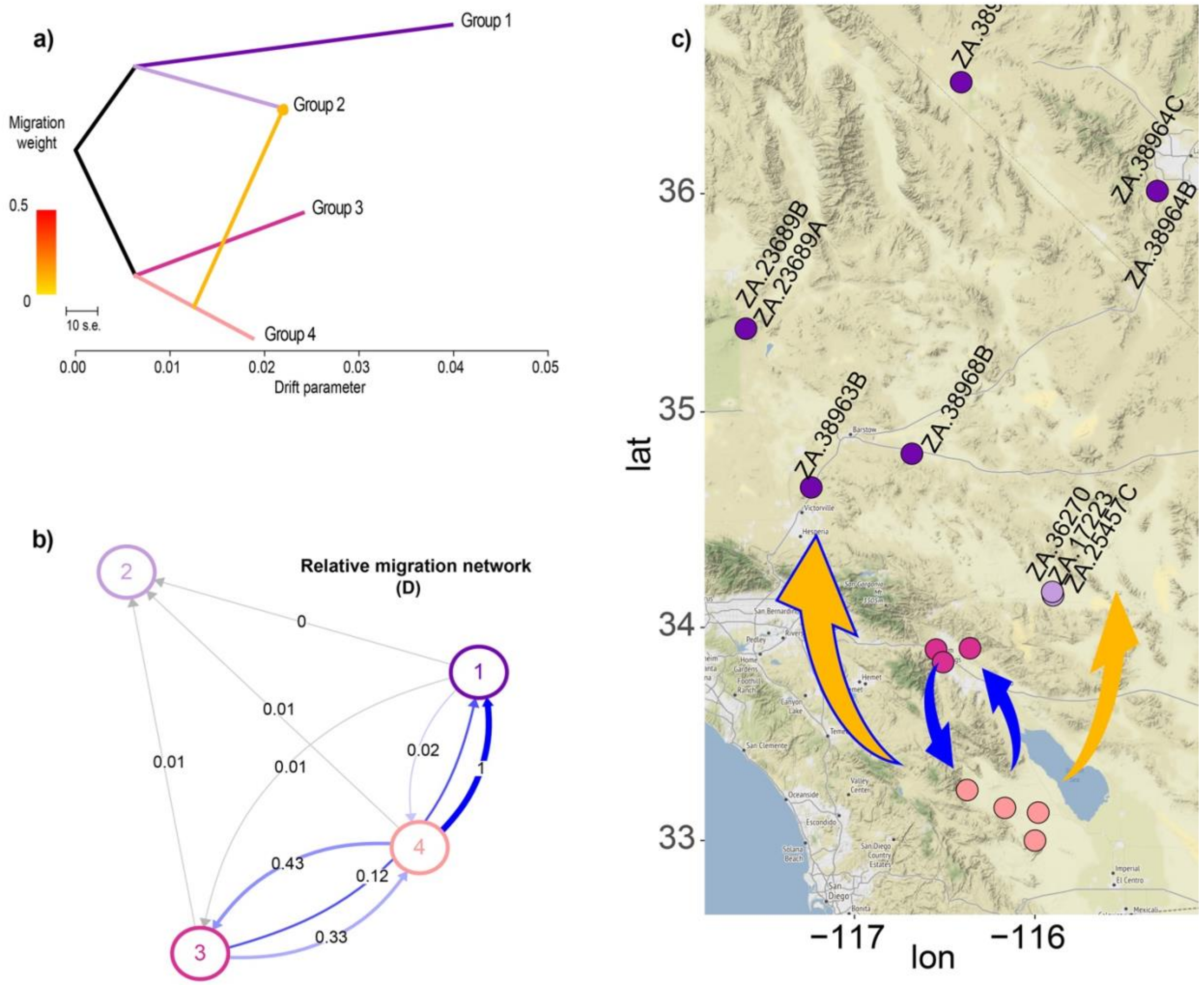

Figure S10. a) Visualization of migration from group 4 towards group 2 as a measure of genetic drift, implemented in a treemix graph considering 100k SNPs and an unrooted topology. b) Relative migration network calculated by the divMigrate function from the R package diveRsity selecting the *Gst* statistics. c) Graphical abstract of the population clusters from the four populations and migration events. Map was created with the R package *ggmap*, a map base from Google Maps, and our locality records for *E. titania*.

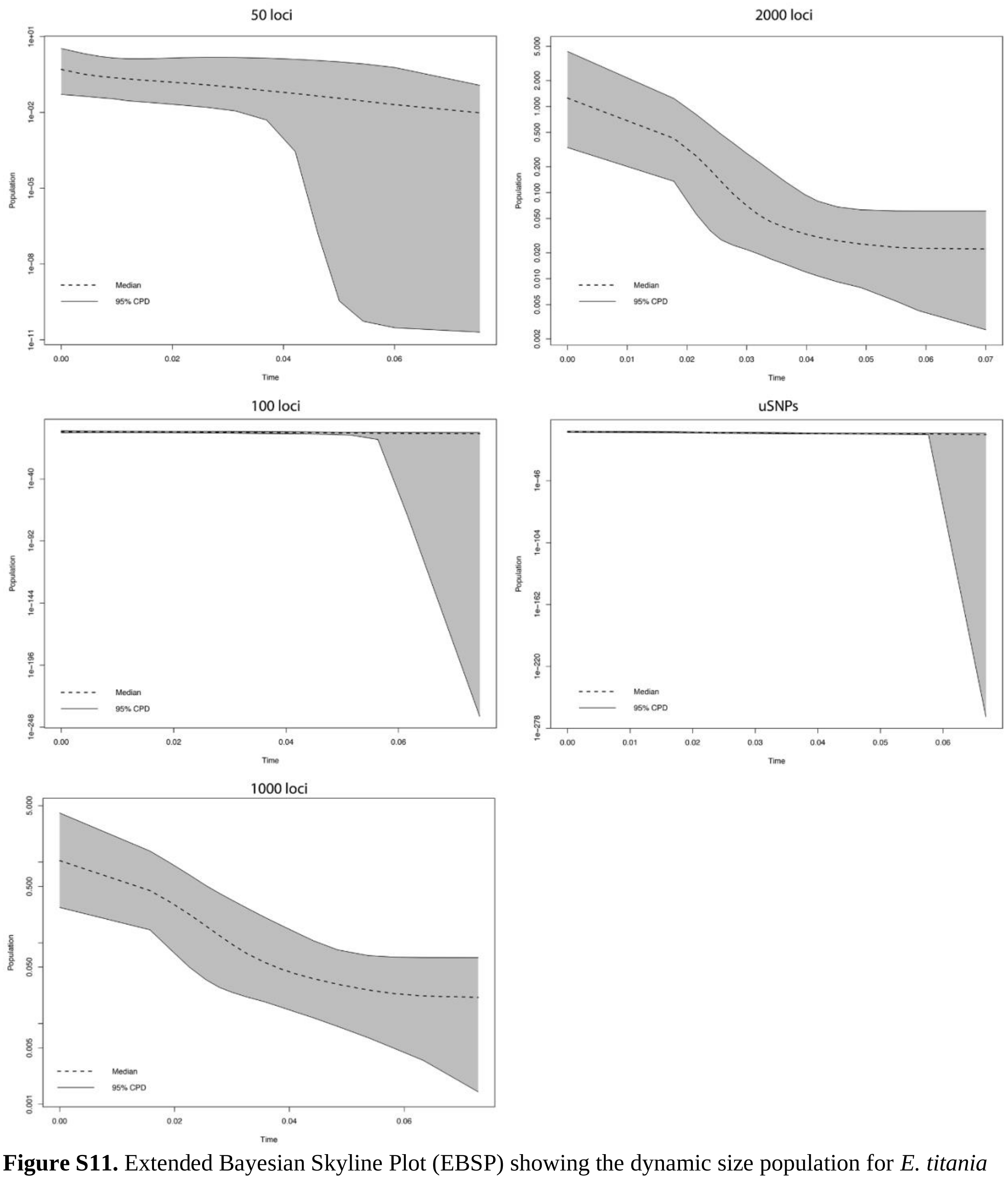

Figure S11. Extended Bayesian Skyline Plot (EBSP) showing the dynamic size population for *E. titania* based on different nucleotide compositions, from 50 nucleotides to 4207 uSNPs. The y-axis indicates an effective population size scaled by mutation rate as a function of time.

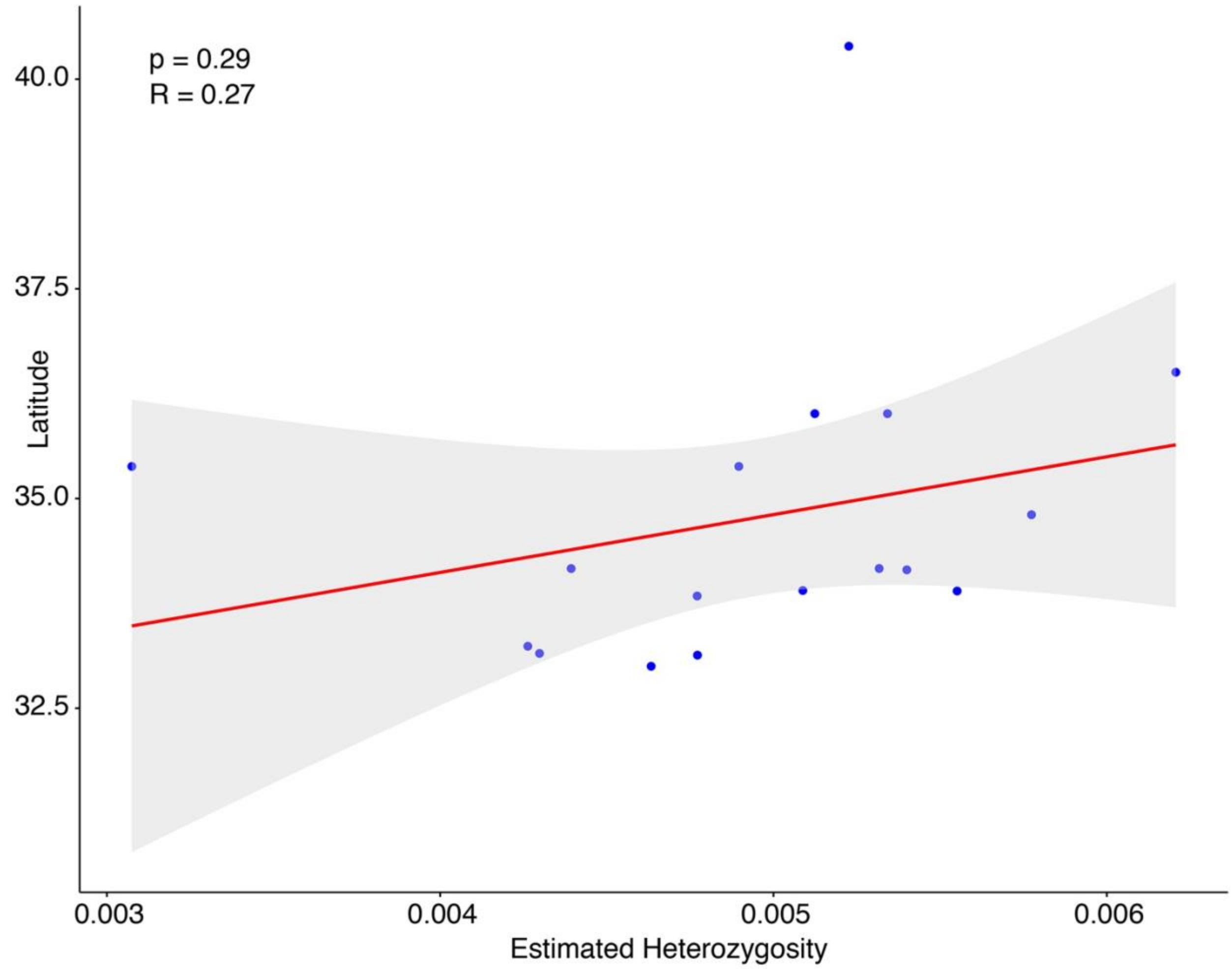

Figure S12. Pearson rank correlation between estimated heterozygosity and latitude. R, correlation coefficient.

Supplementary Tables

Table S1. Description and general properties of the main datasets analyzed

Data set	Number of taxa	Number of loci	Number of sites	Missing data (%)
first matrix (m17)*	66	25,092	384,756	63.93%
m21	42	2,058	521,343	45.12%
m21_SNPs	42	2,058	33,392	45.73%
m21_uSNPs	42	2,058	2,053	45.87%
ec18	23	1,638	452,378	18.63%
ec18_SNPs	23	1,638	21,235	19.97%
ec18_uSNPs	23	1,638	1,637	20.01%
e13	17	4,246	1,153,232	15.79%
e13_SNPs	17	4,246	35,166	17.20%
e13_uSNPs	17	4,246	4,207	17.12%

* This matrix was not analyzed. It served as the starting point for downstream analyses.

Table S2. List of the 68 camel spider samples used in this study. DMNS = Denver Museum of Nature & Science; CAS = California Academy of Sciences; AGS = Coleccion de Zoologia, Universidad Autonoma de Aguascalientes

Specimen #	Species	Collecting date	Stage	Sex	Country	State	County	Locality
DMNS ZA.16323	*Eremocosta bajaensis*	7/1/03	Adult	Male	USA	California	San Diego	Hollenbeck Canyon Wildlife Area
DMNS ZA.17218	*Eremocosta bajaensis*	7/5/05	Adult	Male	USA	California	Riverside	San Jacinto Mnts Hwy 74 Pinyon Pines fire station
DMNS ZA.33051	*Eremocosta bajaensis*	9/12-16/2011	Adult	Female	USA	California	San Diego	San Diego Wild Animal Park [San Diego Zoo Safari Park]
DMNS ZA.33088	*Eremocosta bajaensis*	8/8-13/2010	Adult	Male	USA	California	San Diego	Escondido: San Diego Wild Animal Park [San Diego Zoo Safari Park]
DMNS ZA.33089	*Eremocosta bajaensis*	7/20-24/2009	Adult	Male	USA	California	San Diego	Escondido: San Diego Wild Animal Park [San Diego Zoo Safari Park]
DMNS ZA.36263	*Eremocosta bajaensis*	8/3/11	Adult	Female	USA	California	Riverside	Santa Rosa Mountains, Pinyon Pines on Pinyon Drive, 0.5 miles north of California 74
DMNS ZA.36271	*Eremocosta bajaensis*	9/15/03	Adult	Female	USA	California	San Diego	Alpine: Alpine area
DMNS ZA.33091A	*Eremocosta bajaensis*	8/3-7/2009	Adult	Male	USA	California	San Diego	San Diego National Wildlife Refuge
DMNS ZA.33091B	*Eremocosta bajaensis*	8/3-7/2009	Adult	Male	USA	California	San Diego	San Diego National Wildlife Refuge
DMNS ZA.17214	*Eremocosta calexicensis*	8/31/05	Adult	Male	USA	California	Riverside	Palm Springs: Palm Canyon Dr Junction Bogert Tr
DMNS ZA.30785	*Eremocosta calexicensis*	8/15/13	Adult	Female	USA	California	Riverside	White Water: 13495 Chaparral Rd
DMNS ZA.36257	*Eremocosta calexicensis*	7/13/13	Adult	Female	USA	California	Riverside	Santa Rosa Mountains, west rim of canyon, Pinyon Crest area, 1 mile north of California 74 and Carrizo Road junction
DMNS ZA.36261	*Eremocosta calexicensis*	9/7/10	Adult	Female	USA	California	Imperial	Off California 78, 7/10 mile west of junction with California 86
DMNS ZA.36262	*Eremocosta calexicensis*	9/12/08	Adult	Female	USA	California	San Diego	Borrego Springs: 7/10 mile south of center of Borrego Springs
DMNS ZA.36899	*Eremocosta calexicensis*	9/8/13	Adult	Female	USA	California	Riverside	San Jacinto Mountains: on road to Palm Springs Tramway, 2 7/10 mi W. of Hwy 111
DMNS ZA.40294	*Eremocosta calexicensis*	6/3/17	Adult	Male	USA	Arizona	Mohave	Bull Head City: BLM land just outside of Bull Head City off Backwash RD
AGS 15	*Eremocosta formidabilis*	4/27/13	Adult	Male	MEXICO	Aguascalientes		Pabellon de Arteaga
DMNS ZA.21950	*Eremocosta gigasella*	06/15-16/2009	Adult	Female	USA	Texas	Brewster Co	Dalquest Research site, near shed
DMNS ZA.22964A	*Eremocosta* sp.	06/13-27/2009	Adult	Female	USA	Texas	Brewster Co	Dalquest Research Site, Big Sandy Line 2
DMNS ZA.22964B	*Eremocosta* sp.	06/13-27/2009	Adult	Female	USA	Texas	Brewster Co	Dalquest Research Site, Big Sandy Line 2

DMNS ZA.22969A	*Eremocosta* sp.	06/13-27/2009	Adult	Female	USA	Texas	Brewster Co	Dalquest Research Site, Below Alamo, Line 3
DMNS ZA.22969B	*Eremocosta* sp.	06/13-27/2009	Adult	Female	USA	Texas	Brewster Co	Dalquest Research Site, Below Alamo, Line 3
DMNS ZA.22970A	*Eremocosta* sp.	06/13-27/2009	Adult	Female	USA	Texas	Brewster Co	Dalquest Research Site, Below Alamo, Line 3
DMNS ZA.22970B	*Eremocosta* sp.	06/13-27/2009	Adult	Female	USA	Texas	Brewster Co	Dalquest Research Site, Below Alamo, Line 3
DMNS ZA.22970C	*Eremocosta* sp.	06/13-27/2009	Adult	Female	USA	Texas	Brewster Co	Dalquest Research Site, Below Alamo, Line 3
DMNS ZA.26873C	*Eremocosta* sp.	07/23/10-08/10/10	Adult	Female	USA	Texas	Brewster Co	DDRS below Alamo Spring
DMNS ZA.26873D	*Eremocosta* sp.	07/23/10-08/10/10	Adult	Female	USA	Texas	Brewster Co	DDRS below Alamo Spring
DMNS ZA.27626A	*Eremocosta* sp.	9/4/11	Adult	Female	USA	Texas	Brewster Co	Dalquest Research Site
DMNS ZA.21079	*Eremocosta striata*	7/30/08	Adult	Male	USA	Arizona	Santa Cruz	E. of Amado along Hopkins Mountain Rd.; between the 2-5 Km signs.
DMNS ZA.25446	*Eremocosta striata*	7/28/09	Adult	Male	USA	Arizona	Santa Cruz	Hopkins Mountain Rd, toward Whipple Observatory
DMNS ZA.25451	*Eremocosta striata*	7/9/20	Adult	Female	USA	Arizona	Santa Cruz	Esplendor Resort, 1069 Camino Caralampi, Rio Rico
DMNS ZA.25452	*Eremocosta striata*	7/28/09	Adult	Male	USA	Arizona	Santa Cruz	Hopkins Mountain Rd
DMNS ZA.25453	*Eremocosta striata*	7/28/09	Adult	Male	USA	Arizona	Santa Cruz	Hopkins Mountain Rd.
DMNS ZA.38835	*Eremocosta striata*	7/12/18	Adult	Male	USA	Arizona	Pima	Catalina State Park Campgrounds A & B
CAS 9033523	*Eremocosta striata*	8/9/78	Adult	Male	MEXICO	Sonora		8 km s. Benjamin Hill
DMNS ZA.21706A	*Eremocosta striata*	7/31/08	Adult	Male	USA	Arizona	Santa Cruz	Mt. Hopkins Road, 1500 ft uphill of Amado Montosa Rd
DMNS ZA.21706B	*Eremocosta striata*	7/31/08	Adult	Female	USA	Arizona	Santa Cruz	Mt. Hopkins Road, 1500 ft uphill of Amado Montosa Rd
DMNS ZA.25448A	*Eremocosta striata*	7/9/20	Adult	Male	USA	Arizona	Santa Cruz	Mt. Hopkins Rd
DMNS ZA.25448B	*Eremocosta striata*	7/9/20	Adult	Female	USA	Arizona	Santa Cruz	Mt. Hopkins Rd
DMNS ZA.28605A	*Eremocosta striata*	7/24-25/2011	Adult	Female	USA	Arizona	Yavapai	Cornville:
DMNS ZA.28605B	*Eremocosta striata*	7/24-25/2011	Adult	Female	USA	Arizona	Yavapai	Cornville:
DMNS ZA.17215	*Eremocosta titania*	04/19-20/2006	Adult	Male	USA	California	Riverside Co	Coachella Valley, Palm Springs Amtrak 1/2 mi S junct Indian Cyn Dr & Hwy I-10

DMNS ZA.17222	*Eremocosta titania*	9/2/06	Adult	Male	USA	California	Riverside Co	Coachella Valley NE Edge of Palm Springs jnctn Airport Tachevah Dr just E jnctn Hwy 111
DMNS ZA.17223	*Eremocosta titania*	07/30-31/2006	Adult	Male	USA	California	San Bernardino Co	Wonder Valley Amber Rd 8.5 mi E Twenty Nine Palms at Adobe Rd jnctn
DMNS ZA.17224	*Eremocosta titania*	9/2/06	Adult	Female	USA	California	Riverside Co	Coachella Valley NE edge of Palm Springs intersection Airport and Achevah Dr just N of jnctn Why 111
DMNS ZA.23484	*Eremocosta titania*	9/21/09	Adult	Male	USA	California	Imperial Co	Hwy 78 at Junct. San Felipe Creek 10.5 mi. W. Junct. Hwy 86
DMNS ZA.33238	*Eremocosta titania*	8/25/13	Adult	Male	USA	California	Imperial	cc-11-37-9
DMNS ZA.36256	*Eremocosta titania*	5/18/09	Adult	Female	USA	California	Riverside	Coachella Valley/Sky Valley, near junction of Dillon and Aurora roads
DMNS ZA.36259	*Eremocosta titania*	9/7/10	Adult	Female	USA	California	Imperial	Off California 78, 9 miles west of junction with California 86
DMNS ZA.36260	*Eremocosta titania*	6/7/08	Adult	Female	USA	California	San Diego	Ocotillo Wells ranger station, north of California 78
DMNS ZA.36268	*Eremocosta titania*	9/2/06	Adult	Female	USA	California	Riverside	East side of Palm Springs Int'l. Airport, on East Tachevah Drive, one/tenth mile west of California 111
DMNS ZA.36269	*Eremocosta titania*	10/8/12	Adult	Female	USA	California	San Diego	1.5 miles south of center of Borrego Springs
DMNS ZA.36270	*Eremocosta titania*	7/4/10	Adult	Female	USA	California	San Bernardino	29 Palms: Amboy Road 8 1/2 miles east of 29 Palms (Adobe Road)
DMNS ZA.38969	*Eremocosta titania*	07/30-31/2018	Adult	Male	USA	Nevada	Nye	E Mecca Rd, N. of Longstreet Casino
DMNS ZA.38971	*Eremocosta titania*	08/01-02/2018	Adult	Male	USA	California	San Bernardino	N Victorville, Powerline Rd.; 34.6511 - 117.239968
DMNS ZA.40671	*Eremocosta titania*	6/4/17	Adult	Male	USA	Nevada	Clark	public land just south Lake Mead Recreation Area, West Laughlin Landfill
CAS 9033514	*Eremocosta titania*	7/22/85	Adult	Female	USA	Nevada	Clark	Las Vegas:
DMNS ZA.23689A	*Eremocosta titania*	6/15/10	Adult	Female	USA	California	San Bernardino	BLM land in Mojave desert off Trona Rd North of Red Mountain and west of Golden Valley Wilderness
DMNS ZA.23689B	*Eremocosta titania*	6/15/10	Adult	Female	USA	California	San Bernardino	BLM land in Mojave desert off Trona Rd North of Red Mountain and west of Golden Valley Wilderness
DMNS ZA.25457C	*Eremocosta titania*	5/17/07	Adult	Male	USA	California	San Bernardino	Wonder Valley Amboy Rd, 8.5 mi E Twenty Nine Palms (at Adobe Rd. junction); 34.1657 - 115.9037
DMNS ZA.33963B	*Eremocosta titania*	08/01-02/2018	Adult	Female	USA	California	San Bernardino	N Victorville, Powerline Rd.
DMNS ZA.38964B	*Eremocosta titania*	07/31/18 - 08/01/2018	Adult	Male	USA	Nevada	Clark	Las Vegas: S Hualapai Way
DMNS ZA.38964C	*Eremocosta titania*	07/31/18 - 08/01/2018	Adult	Male	USA	Nevada	Clark	Las Vegas: S Hualapai Way
DMNS ZA.38968B	*Eremocosta titania*	07/29-30/2018	Adult	Female	USA	California	San Bernardino	Newberry Springs: Newberry Springs, Newberry Rd.

DMNS ZA.38968C	*Eremocosta titania*	07/29-30/2018	Juvenile	Female	USA	California	San Bernardino	Newberry Springs: Newberry Springs, Newberry Rd.
CAS 9033928C	*Eremocosta titania*	7/15/87	Adult	Female	USA	Nevada	Clark	Las Vegas:
DMNS ZA.28289A	*Hemerotrecha branchi*	7/25/11	Adult	Male	USA	California	San Bernardino	29 Palms, Amboy Road, 1 mile East junction Adobe Rd.
DMNS ZA.28289B	*Hemerotrecha branchi*	7/25/11	Adult	Male	USA	California	San Bernardino	29 Palms, Amboy Road, 1 mile East junction Adobe Rd.
MRG 1811	*Ammotrechula sp.*	07/31/18 - 08/01/2018	Adult	Unk	USA	Nevada	Clark	Las Vegas: S Hualapai Way

Table S3. Assembly statistics of the 68 camel spider specimens using ipyrad

DMNS ZA#	Qbit from Extraction (ng/uL)	reads_raw	reads_passed_filter	refseq_mapped_reads	refseq_unmapped_reads	clusters_total	clusters_hidepth	hetero_est	error_est	reads_consens	loci_in_assembly
16323	2.76	Didn't pass the filters from step 5									
17214	8.7	596155	595390	558564	36826	88660	21221	0.005048	0.001467	21216	769
17215	6.52	21100460	21084752	12463804	8620948	380232	104757	0.005551	0.001015	104431	1467
17218	5.06	966892	966113	898447	67666	92284	27907	0.003908	0.001481	27893	863
17222	5.74	51890	51832	28439	23393	9249	1053	0.003951	0.00321	1053	51
17223	4.08	257015	256753	173346	83407	30916	5612	0.004393	0.001916	5609	239
17224	7.32	Didn't pass the filters from step 5									
21079	1.19	1028822	1027730	976760	50970	106644	28997	0.004663	0.001393	28991	850
21950	6.04	316067	315809	307838	7971	45820	11743	0.00473	0.001412	11741	466
23484	4.36	10055656	10049595	8749405	1300190	229913	69429	0.004633	0.000851	69328	1421
25446	3.08	15389	15332	14669	663	6270	445	0.003674	0.001658	445	9
25451	3.16	2632020	2619964	1993441	626523	138347	73244	0.004404	0.001171	73217	1139
25452	0.82	1665	1660	1651	9	522	42	0.002853	0.002009	42	1
25453	1.21	60897	60584	57424	3160	11563	2122	0.003178	0.00129	2121	56
30785	0.24	2045	2037	1529	508	308	50	0.000685	0.0028	50	1
33051	1.88	35479	35415	33945	1470	5636	1102	0.003662	0.001463	1102	34
33088	3.72	26669	26563	24838	1725	5832	933	0.004563	0.00185	933	17
33089	1.73	6342	6217	5672	545	1996	200	0.005708	0.001901	200	3
33238	10.8	281347	280687	226096	54591	13818	3357	0.002365	0.000792	3357	38

36256	5.88	7750136	7743587	5416786	2326801	202004	75418	0.005088	0.001121	75325	1416
36257	3.6	13738010	13720288	12331715	1388573	331207	101744	0.004574	0.000703	101584	1430
36259	10.2	15817325	15803601	11278956	4524645	321317	100245	0.004772	0.000974	99991	1466
36260	9.32	13101338	13084014	8992220	4091794	340733	121299	0.004298	0.001005	121146	1433
36261	0.38	2543572	2540700	2332129	208571	122143	49675	0.003979	0.001104	49638	1057
36262	3.12	16762012	16720891	13736073	2984818	381671	109449	0.00422	0.000704	109219	1433
36263	2.6	31014	30988	30039	949	3640	867	0.002835	0.000919	867	21
36268	0.26	4238270	4234236	2500169	1734067	127107	53174	0.004771	0.001323	53119	1091
36269	7.38	2847207	2829663	1737542	1092121	59994	13313	0.004263	0.000617	13272	130
36270	2.16	27055859	27034041	18585301	8448740	507899	116113	0.0054	0.000759	115711	1471
36271	2.34	13836386	13822527	10139602	3682925	319816	83528	0.001706	0.000981	83304	368
36899	0.7	1965027	1963685	1890075	73610	94178	33884	0.003406	0.001065	33866	978
38835	1.82	252843	252390	217690	34700	48129	9469	0.003327	0.001551	9469	324
38969	1.87	22421422	22397451	15855162	6542289	543559	122593	0.006207	0.000907	122270	1447
38971	2.24	45452	45342	24268	21074	3855	146	0.024113	0.008062	146	0
40294	3.32	344337	343671	320012	23659	71822	14794	0.00495	0.001817	14789	521
40671	1.75	Didn't pass the filters from step 5									
9033514	2.54	197351	195187	130487	64700	24147	1033	0.012759	0.00749	1026	17
9033523	2.78	782	765	765	0	683	1	0.027077	0.007179	1	0
21706A	3.74	330228	329882	314094	15788	31295	8516	0.003122	0.001185	8515	232
21706B	4.8	43290	43182	40936	2246	7707	1068	0.00297	0.000918	1068	22
22964A	0.23	215239	214936	198754	16182	15964	3316	0.003426	0.001152	3312	48

22964B	0.71	3911	3904	3888	16	3040	20	0.01441	0.004624	20	1
22969A	6.46	13871652	13856189	12244747	1611442	377117	103207	0.003602	0.000811	103049	872
22969B	1.15	1732956	1730010	1539632	190378	132089	41309	0.004317	0.001347	41290	642
22970A	1.49	1506998	1500547	1302511	198036	155277	41566	0.004464	0.001335	41539	610
22970B	1.23	2000	1994	1493	501	1049	5	0.02841	0.013088	5	1
22970C	2.4	46869	46776	17274	29502	3843	112	0.025374	0.008063	111	0
23689A	9.62	500094	499672	452540	47132	32358	10333	0.003075	0.001171	10329	407
23689B	11	5910861	5906814	5361270	545544	186916	59955	0.004896	0.000973	59909	1342
25448A	1.45	4774	4656	4063	593	2358	68	0.00884	0.00168	68	2
25448B	1.48	891	886	885	1	500	13	0.004111	0.001057	13	0
25457C	7.46	24925557	24905450	21847718	3057732	551446	129457	0.005317	0.000697	129141	1465
26873C	4.74	374758	374325	353962	20363	33214	8402	0.002445	0.001047	8398	122
26873D	6.9	20656821	20637023	18098237	2538786	492749	128971	0.003467	0.00068	128713	848
27626A	4.34	27120	27089	26879	210	3348	658	0.003158	0.0011	658	9
28289A	7.96	19980046	19942788	16658082	3284706	470612	101606	0.00473	0.000607	101433	140
28289B	5.14	5300722	5296952	4922580	374372	174808	63059	0.004715	0.000855	63002	137
28605A	0.75	9276	9232	8017	1215	3394	188	0.003823	0.002503	188	2
28605B	4.52	3817946	3814826	3625557	189269	152740	64564	0.001731	0.00097	64535	1134
33091A	5.4	167420	167263	154414	12849	24692	4351	0.0043	0.001492	4349	128
33091B	5.44	141609	141197	130186	11011	25289	3616	0.004543	0.001536	3616	80
33963B	9.24	601713	601178	510445	90733	73750	18654	0.005226	0.00201	18650	720
38964B	6.78	31164921	31138364	26398533	4739831	644582	106901	0.005342	0.000572	106360	1469

38964C	5.88	21024092	21008883	18129637	2879246	476100	105229	0.005124	0.000597	104965	1469
38968B	7.08	17241073	17222101	13851851	3370250	393859	101919	0.005774	0.000816	101701	1473
38968C	6.52	201404	201238	195652	5586	48544	9607	0.002928	0.000981	9607	63
9033928C	1.28	164	164	164	0	149	1	0.024742	0.015716	1	0
A6515	5.92	649327	635120	562724	72396	68542	27138	0.005175	0.001571	27122	170

Table S4. Ecological region areas designated to each sample

DMNS ZA#	Species	Area	Code
17218	*Eremocosta bajaensis*	10.2.2 Sonoran Desert	B
33051	*Eremocosta bajaensis*	11.1.1 California Coastal Sage, Chaparral, and Oak Woodland	D
33088	*Eremocosta bajaensis*	10.2.2 Sonoran Desert	B
36271	*Eremocosta bajaensis*	11.1.1 California Coastal Sage, Chaparral, and Oak Woodland	D
33091A	*Eremocosta bajaensis*	11.1.1 California Coastal Sage, Chaparral, and Oak Woodland	D
33091B	*Eremocosta bajaensis*	11.1.1 California Coastal Sage, Chaparral, and Oak Woodland	D
17214	*Eremocosta calexicensis*	10.2.2 Sonoran Desert	B
36257	*Eremocosta calexicensis*	10.2.2 Sonoran Desert	B
36261	*Eremocosta calexicensis*	10.2.2 Sonoran Desert	B
36262	*Eremocosta calexicensis*	10.2.2 Sonoran Desert	B
36899	*Eremocosta calexicensis*	10.2.2 Sonoran Desert	B
40294	*Eremocosta calexicensis*	10.2.1 Mojave Basin and Range	A
21950	*Eremocosta gigasella*	10.2.4 Chihuahuan Desert	C
22964A	*Eremocosta* sp.	10.2.4 Chihuahuan Desert	C
22969A	*Eremocosta* sp.	10.2.4 Chihuahuan Desert	C
22969B	*Eremocosta* sp.	10.2.4 Chihuahuan Desert	C
22970A	*Eremocosta* sp.	10.2.4 Chihuahuan Desert	C
26873C	*Eremocosta* sp.	10.2.4 Chihuahuan Desert	C
26873D	*Eremocosta* sp.	10.2.4 Chihuahuan Desert	C
21079	*Eremocosta striata*	10.2.2 Sonoran Desert	B
25451	*Eremocosta striata*	12.1.1 Madrean Archipelago	E
25453	*Eremocosta striata*	12.1.1 Madrean Archipelago	E
38835	*Eremocosta striata*	12.1.1 Madrean Archipelago	E
21706A	*Eremocosta striata*	10.2.2 Sonoran Desert	B
28605B	*Eremocosta striata*	13.1.1 Arizona/New Mexico Mountains	F
17215	*Eremocosta titania*	10.2.2 Sonoran Desert	B
17223	*Eremocosta titania*	10.2.1 Mojave Basin and Range	A
23484	*Eremocosta titania*	10.2.2 Sonoran Desert	B
36256	*Eremocosta titania*	10.2.2 Sonoran Desert	B
36259	*Eremocosta titania*	10.2.2 Sonoran Desert	B
36260	*Eremocosta titania*	10.2.2 Sonoran Desert	B
36268	*Eremocosta titania*	10.2.2 Sonoran Desert	B
36269	*Eremocosta titania*	10.2.2 Sonoran Desert	B
36270	*Eremocosta titania*	10.2.1 Mojave Basin and Range	A
38969	*Eremocosta titania*	10.2.1 Mojave Basin and Range	A
23689A	*Eremocosta titania*	10.2.1 Mojave Basin and Range	A
23689B	*Eremocosta titania*	10.2.1 Mojave Basin and Range	A
25457C	*Eremocosta titania*	10.2.1 Mojave Basin and Range	A

33963B	*Eremocosta titania*	10.2.1 Mojave Basin and Range	A
38964B	*Eremocosta titania*	10.2.1 Mojave Basin and Range	A
38964C	*Eremocosta titania*	10.2.1 Mojave Basin and Range	A
38968B	*Eremocosta titania*	10.2.1 Mojave Basin and Range	A
38968C	*Eremocosta titania*	10.2.1 Mojave Basin and Range	A

Table S5. Comparative scores of the six types of biogeographical models tested on Eremocosta phylogeny using RASP

Molecular dating using Cushing et al. (2015) calibration points (D18)

Model	LnL	AICc	AICc_wt
DEC	-41.14	86.62	0.0014
DEC+J	**-34.13**	**74.97**	**0.47**
DIVALIKE	-40.16	84.67	0.0036
DIVALIKE+J	-34.15	75	0.46
BAYAREALIKE	-56.35	117	3.40E-10
BAYAREALIKE+J	-36.02	78.74	0.071

Molecular dating using our beast analysis calibration points (D5)

	LnL	AICc	AICc_wt
DEC	-41.24	86.82	0.0012
DEC+J	**-34.13**	**74.97**	**0.47**
DIVALIKE	-40.27	84.87	0.0033
DIVALIKE+J	-34.15	75	0.46
BAYAREALIKE	-56.44	117.2	3.10E-10
BAYAREALIKE+J	-36.02	78.74	0.071
